# Resistance Mechanism and Physiological Effects of Microcin Y in Salmonella enterica subsp. *enterica* Serovar Typhimurium

**DOI:** 10.1128/spectrum.01859-22

**Published:** 2022-12-01

**Authors:** Yu Han, Yu Li, Zhiwei Zeng, Wenjing Li, Saixiang Feng, Weisheng Cao

**Affiliations:** a College of Veterinary Medicine, South China Agricultural University, Guangzhou, China; b Key Laboratory of Zoonosis Prevention and Control of Guangdong Province, Guangzhou, China; c Key Laboratory of Zoonosis of Ministry of Agriculture and Rural Affairs, Guangzhou, China; d Key Laboratory of Veterinary Vaccine Innovation of the Ministry of Agriculture and Rural Affairs, Guangzhou, China; e National and Regional Joint Engineering Laboratory for Medicament of Zoonosis Prevention and Control, Guangzhou, China; Health Canada

**Keywords:** *Salmonella* Typhimurium, lasso peptide, MccY, resistance, Ton system, virulence, *Salmonella*, drug resistance mechanisms, virulence factors

## Abstract

Salmonella bacteria pose a significant threat to animal husbandry and human health due to their virulence and multidrug resistance. The lasso peptide MccY is a recently discovered antimicrobial peptide that acts against various serotypes of Salmonella. In this study, we further explore the resistance mechanism and activity of MccY. Mutants of Ton system genes, including *tonB*, *exbB*, and *exbD*, in Salmonella enterica subsp. *enterica* serovar Typhimurium were constructed, and the MICs to MccY exhibited significant increases in these deletion mutants compared to the MIC of the parent strain. Subsequently, MccY resistance was quantitatively analyzed, and these mutants also showed greatly reduced rates of killing, even with a high concentration of MccY. In addition, a minimal medium with low iron environment enhanced the sensitivity of these mutants to MccY. Measurements of a series of physiological indicators, including iron utilization, biofilm formation, and motility, demonstrated that MccY may decrease the virulence of *S.* Typhimurium. Transcriptomic analysis showed that iron utilization, biofilm formation, flagellar assembly, and virulence-related genes were downregulated to varying degrees when *S.* Typhimurium was treated with MccY. In conclusion, deletion of Ton system genes resulted in resistance to MccY and the susceptibility of these mutants to MccY was increased and differed under a low-iron condition. This lasso peptide can alter multiple physiological properties of *S.* Typhimurium. Our study will contribute to improve the knowledge and understanding of the mechanism of MccY resistance in Salmonella strains.

**IMPORTANCE** The resistance of Salmonella to traditional antibiotics remains a serious challenge. Novel anti-Salmonella drugs are urgently needed to address the looming crisis. The newly identified antimicrobial peptide MccY shows broad prospects for development and application because of its obvious antagonistic effect on various serotypes of Salmonella. However, our previous study showed that the peptide could confer resistance to Salmonella by disrupting the receptor gene *fhuA*. In this study, we further explored the potential resistance mechanism of MccY and demonstrated the importance of the Salmonella Ton complex for MccY transport. Disruption in Ton system genes resulted in *S.* Typhimurium resistance to this peptide, and MccY could alter multiple bacterial physiological properties. In summary, this study further explored the resistance mechanism and antibacterial effect of MccY in *S.* Typhimurium and provided a scientific basis for its development and application.

## INTRODUCTION

Salmonella is an important foodborne pathogen that can spread to humans through animals and the food chain (1). It can cause serious harm to animal production and human health, with important public health significance. Salmonella enterica subsp. *enterica* serovar Typhimurium is one of the most common serotypes causing foodborne diseases. It possesses fimbria involved in biofilm formation, flagella related to motility, the type III secretion systems (T3SS I and II) encoded by virulence islands Salmonella pathogenicity island 1 (SPI-1) and SPI-2, and other virulence-related factors, such as iron utilization genes, and therefore, it has a relatively high pathogenicity (2–4). *S.* Typhimurium can cause a variety of diseases, from gastroenteritis to systemic infection, in different hosts, including humans and animals (5). Humans are mainly infected with *S.* Typhimurium by eating contaminated food of animal origin (6). Under such conditions, animal food producers and processors must employ efficient preventive strategies to control Salmonella (including the Typhimurium serotype) throughout the supply chain (7).

With the wide application of antibiotics in the clinic, strains of Salmonella with resistance to antibiotics are now widespread all over the world (8). Multidrug-resistant (MDR) strains of Salmonella, which are resistant to three or more antibiotics, have been observed. More alarming than the emergence of the MDR strains is the recent emergence of extensively drug-resistant (XDR) Salmonella strains (9), such as the recently reported strains of S. enterica subspecies enterica serovar Indiana (10) and *S.* Typhimurium (11) that are resistant to almost all types of antibiotics. Thus, the rapid emergence and increasing severity of antibiotic resistance have led to an urgent need for novel antimicrobial strategies.

Microcins are ribosomally synthesized antimicrobial peptides produced by Gram-negative bacteria from the family *Enterobacteriaceae* that exert antagonistic activity against closely related bacteria (12, 13). According to previous studies, most microcins use one of the two energy transfer systems, the Ton system and the Tol-Pal system, to transfer energy and hijack the outer membrane protein FhuA, which is the natural siderophore receptor of Salmonella, and then enter the target cell through the inner membrane transporter SbmA (14–16). Both the Ton system and the Tol-Pal system are multisubunit membrane protein complexes. The former includes three proteins, namely, TonB, ExbB, and ExbD, and the latter includes five proteins, namely, TolA, TolB, TolQ, TolR, and Pal. These two complexes, which are widely present in Gram-negative bacteria, can convert plasma membrane proton kinetic energy into usable energy, thereby completing the transmembrane transport of substances (17). Due to their high thermal stability, chemical stability, proteolytic stability, and low-dose bactericidal biological activity, microcins have become potential alternatives to antibiotics for increasing food security through the control of food pathogens and spoilage microorganisms (18).

The recently described class I microcin MccY is a plasmid-encoded peptide composed of 21 l-amino acids that has antibacterial effects on many Salmonella strains of different serotypes, including *S.* Typhimurium (19). Because of its lasso structure, it also belongs to the lasso peptide family, with a stable structure and properties. This peptide has the potential to be used in food preservation and as an animal feed additive to reduce the possibility of Salmonella transmission from the food chain to humans.

**FIG 1:**
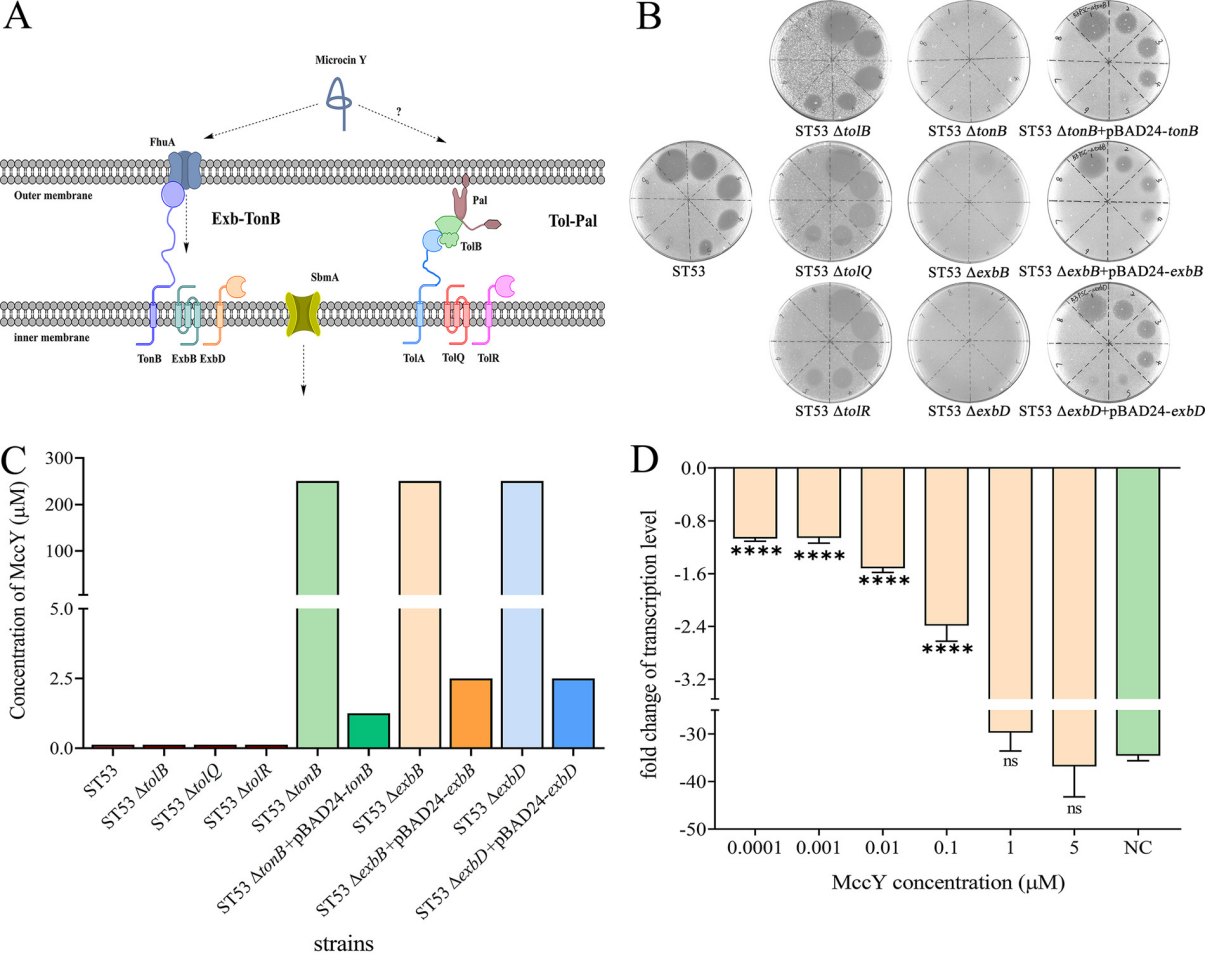
Sensitivity of the *tonB*, *exbB*, and *exbD* mutants to MccY. (A) Schematic diagram of the MccY internalization pathways. (B) Spot-on-lawn assay of MccY against ST53, ST53 Δ*tolB*, ST53 Δ*tolQ*, ST53 Δ*tolR*, ST53 Δ*tonB*, ST53 Δ*exbB*, ST53 Δ*exbD*, and the corresponding complementation strains. Serial dilutions from 250 μM (spot 1) to 0.125 μM (spot 8) were tested. (C) MIC comparison of ST53, ST53 Δ*tolB*, ST53 Δ*tolQ*, ST53 Δ*tolR*, ST53 Δ*tonB*, ST53 Δ*exbB*, ST53 Δ*exbD*, and the corresponding complementation strains. (D) RNA polymerase inhibition *in vitro*. MccY was tested at concentrations of 5, 1, 0.1, 0.01, 0.001, and 0.0001 μM in triplicate, with the transcription level of the *rpsM* gene of MG1655 in each group determined using qRT-PCR. Based on the results for the positive-control group without MccY, the fold change of the transcription level of the *rpsM* gene in each group was calculated and compared with the transcription level of the negative-control group (the same reaction conditions without E. coli RNA polymerase). Error bars show standard deviations. ****, *P* < 0.0001; ns, nonsignificant.

According to our previous study, the receptor FhuA and the transporter SbmA are essential for MccY internalization (19), but which energy transfer system is involved in the process of MccY entering bacteria remains unclear (Fig. 1A). Moreover, bacteria have already evolved several mechanisms to resist killing by microcins, and the ability to survive microcin exposure may be a virulence factor of several human pathogens (20). For example, MccF can cleave an amide bond between the C-terminal aspartate and the nucleotide moiety to detoxify microcin C, thereby conferring microcin C resistance (21). A mutation produced in the conserved size of the *rpoC* gene can make Escherichia coli resistant to microcin J25 (22). It is known that disruptions of FhuA and SbmA confer MccY resistance to Salmonella (19). Given the ability of bacteria to adapt to new environments, it seems possible that Salmonella could also select other anti-MccY mechanisms.

Here, through the single-gene knockout of *tonB*, *exbB*, and *exbD* genes in the Ton system, we confirmed the importance of the Ton system in the transport of MccY. Disruption of *tonB*, *exbB*, or *exbD* increased the resistance of *S.* Typhimurium to MccY, and a minimal medium with a low-iron environment enhanced the sensitivity of these mutants to MccY. In addition, a series of physiological indicators, including iron utilization, biofilm formation, and motility, along with transcriptome analysis, showed that treatment with MccY or the deletion of Ton system genes may negatively affect the virulence of *S.* Typhimurium. This study will help us to further understand the resistance mechanism of MccY and to evaluate its application prospects.

## RESULTS

### Disruption of *tonB*, *exbB*, or *exbD* results in MccY resistance.

Microcins reported by previous studies use the Ton or Tol-Pal system to complete transmembrane transport (23–25). To further explore which system is involved in MccY transport, Ton system-related *tonB*, *exbB*, and *exbD* mutants and Tol-Pal system-related *tolB*, *tolQ*, and *tolR* mutants were constructed from ST53 through the λ-red system (Fig. S1 in the supplemental material) (26). Thereafter, the susceptibilities of the mutants to MccY were assayed by spot-on-lawn methods and MIC determination. The results of the spot-on-lawn experiments intuitively reflected the resistance levels of the mutants (Fig. 1B). The MIC values of the parent strain and the *tolB*, *tolQ*, and *tolR* mutants were 0.125 μM, while those of the *tonB*, *exbB*, and *exbD* mutants were 250 μM (Fig. 1C). Notably, the MIC values of only the Ton system-related *tonB*, *exbB*, and *exbD* mutants increased 2,000-fold, which indicated increased resistance to MccY compared with that of the parent strain ST53 and Tol-Pal system-related *tolB*, *tolQ*, and *tolR* mutants. Moreover, recombinant expression plasmids were constructed to obtain the corresponding complementation strains related to the Ton system (Fig. S2). The sensitivity of all the complementation strains to MccY compared with that of the parent strain was partially compensated. In addition, we tested for *in vitro* RNA polymerase (RNAP) inhibition using MccY against E. coli RNAP (Fig. 1D). The result showed that MccY inhibits transcription by bacterial RNA polymerase and that MccY uptake might be the limiting factor to its activity against *Enterobacteriaceae* bacteria. Altogether, these results indicated that the Ton system is essential for MccY transport in Salmonella and that disruption of any one of the three genes in the Ton system can lead to MccY resistance in *S.* Typhimurium.

### Quantification of MccY resistance in Ton system mutants.

**FIG 2:**
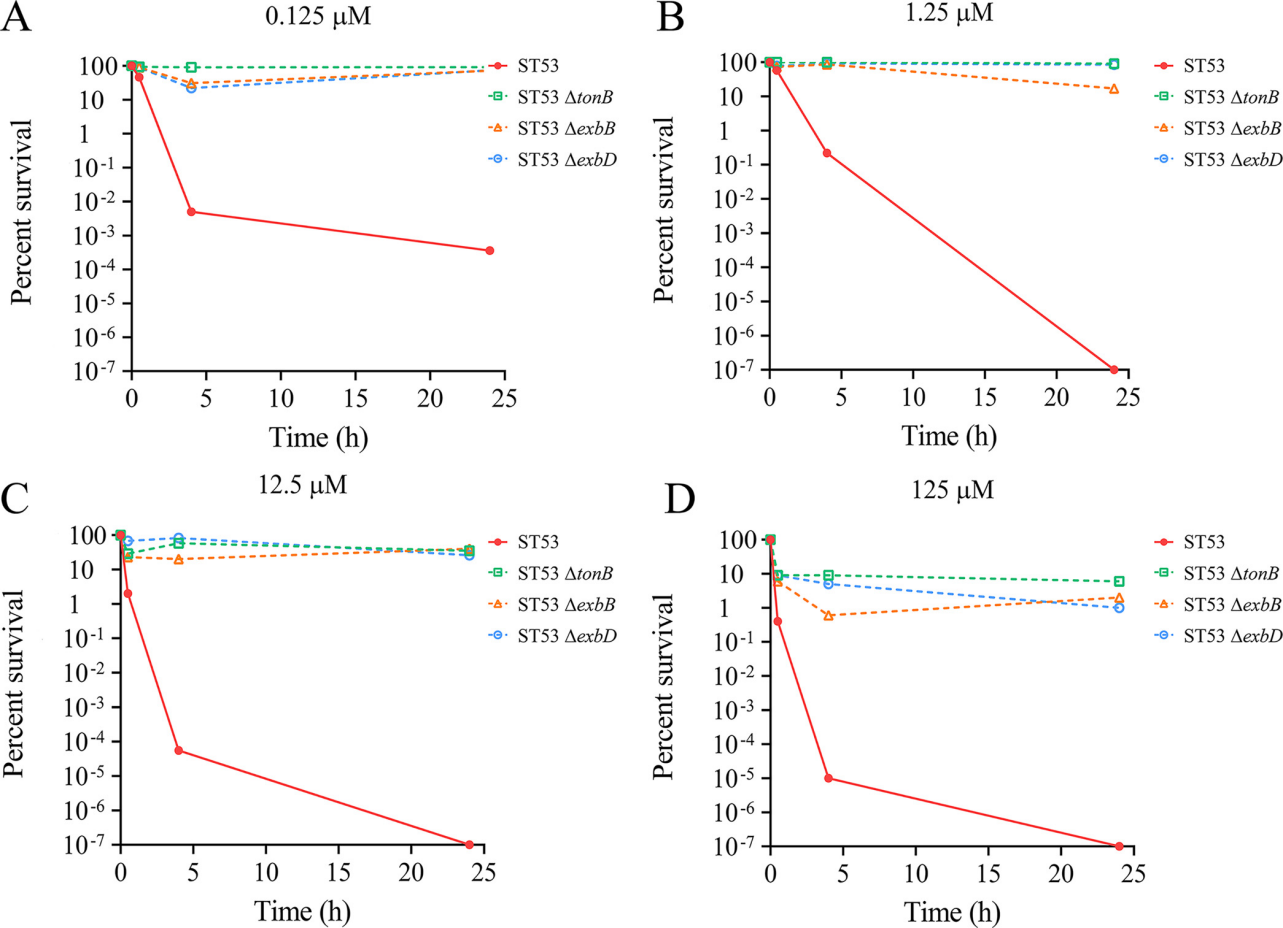
Kill curves for MccY-resistant mutants and the wild type at the following concentrations of MccY: 0.125 μM (A), 1.25 μM (B), 12.5 μM (C) and 125 μM (D). Bacteria were treated with MccY, and the numbers of viable cells in MccY-treated groups and controls without peptide were measured at 0, 0.5, 4, and 24 h. The value of the bacterial survival rate was calculated by dividing the number of colonies in the treatment group by the number of colonies in the control group. The curves represent the survival rates of live bacteria over time relative to that of a control without peptide.

The susceptibilities of the parent strain and the Ton system mutants to MccY were assayed by further examining killing over time at different concentrations of MccY. We plotted the time-kill kinetics (changes in viable bacteria over time) of bacteria at different concentrations of MccY relative to those in a control without the peptide (Fig. 2). For the parent strain, the survival rate of bacteria decreased approximately 5-log in 24 h at the lowest concentration of MccY (0.125 μM) (Fig. 2A), and the survival rate showed a 9-log decrease at the higher concentrations of MccY (1.25, 12.5, and 125 μM) compared with that of the control (Fig. 2B to D). However, the Ton system mutants showed survival rates similar to that of the control at lower concentrations of MccY (0.125, 1.25, and 12.5 μM). After incubation for 4 h at the highest concentration of the peptide (125 μM), the survival rate started to show a 1- to 2-log decrease compared with that of the control. Since no significant decrease in the survival rate of bacteria was observed, the Ton system mutants had a much greater capacity than the parent strain to survive even at higher concentrations of MccY.

### Changes of MccY sensitivity in *S.* Typhimurium under different iron concentrations.

**FIG 3:**
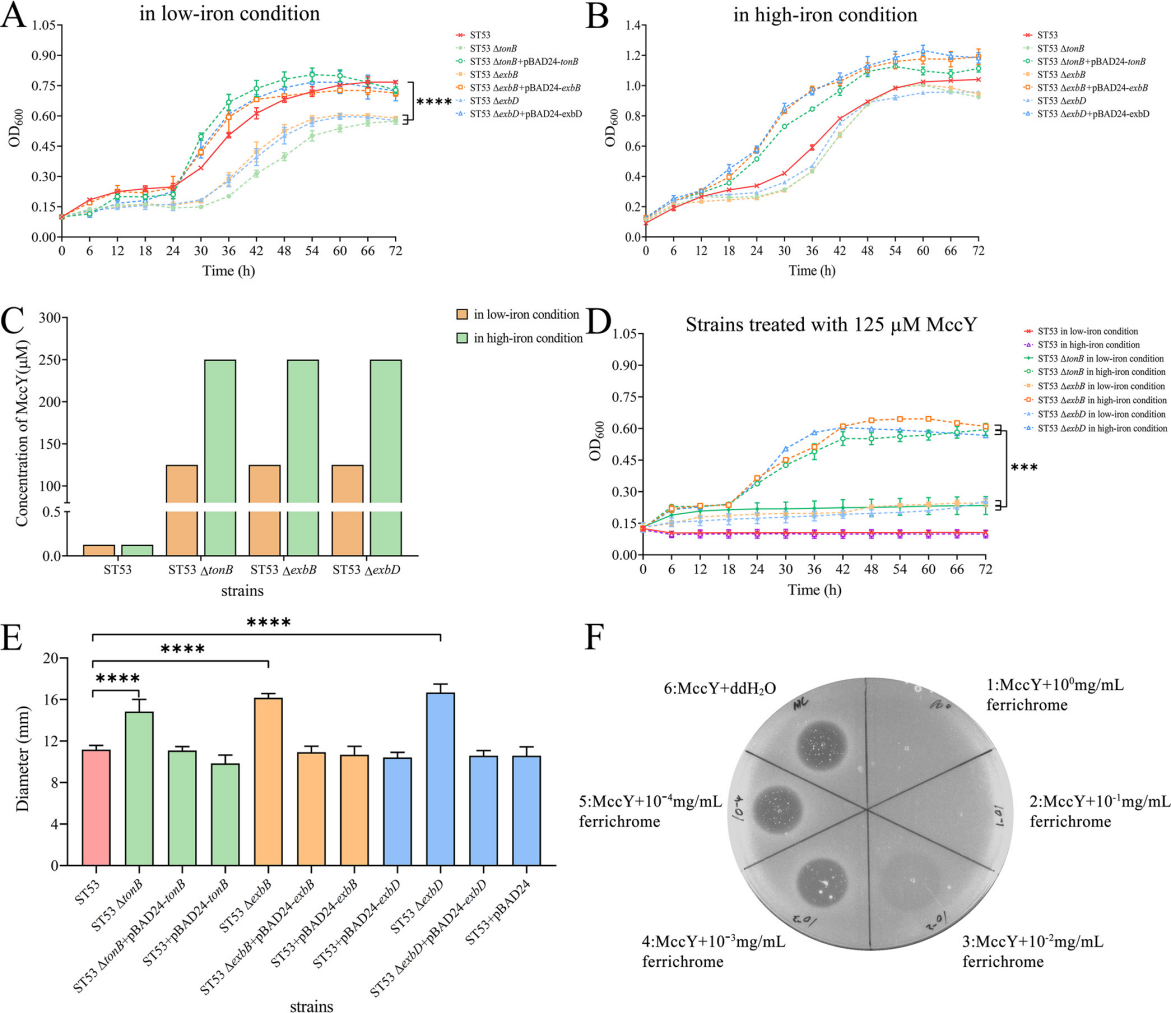
Growth curves and sensitivity of *S.* Typhimurium under low-iron and high-iron conditions. (A and B) The growth curves of the wild type (ST53), the Δ*tonB*, Δ*exbB*, and Δ*exbD* mutants, and the respective complementation strains were assessed in M63 medium (A) and M63 plus FeSO_4_ medium (50 μM) (B). The optical density at 600 nm (OD_600_) was measured every 2 h by using a BioScreen C. The results represent three independent experiments. (C) Comparison of the MIC values for ST53, ST53 Δ*tonB*, ST53 Δ*exbB*, and ST53 Δ*exbD* in M63 medium and M63 plus FeSO_4_ medium (50 μM). (D) Comparison of the growth for ST53, ST53 Δ*tonB*, ST53 Δ*exbB*, and ST53 Δ*exbD* under low-iron (M63 medium) and high-iron (M63 medium plus 50 μM FeSO4) conditions when treated with 125 μM MccY. (E) The chrome azurol S (CAS) assay was used to evaluate the production of siderophores. The blue color turns to yellow-orange haloes when iron is removed from CAS by higher-affinity siderophores. Comparison of the production of siderophores by measuring the diameters of yellow-orange haloes in ST53, ST53 Δ*tonB*, ST53 Δ*exbB*, and ST53 Δ*exbD*. (F) Determination of the competitive effect of ferrichrome and MccY by agar diffusion assay. Ferrichrome was serially diluted from 1 mg/mL (spot 1) to 10^−4 ^mg/mL (spot 5). The mixtures containing 5 μL of various concentrations of ferrichrome and 5 μL of 50 μg/mL MccY were placed at the center of each area of the soft plate. A negative control without ferrichrome was established with ddH_2_O (spot 6). Error bars show standard deviations. ***, *P < *0.001; ****, *P < *0.0001.

Iron is an essential element for Salmonella, while the acquisition of sufficient amounts of iron is difficult in many environments, including the intestinal tract, where the bacteria usually reside (27). Moreover, MccJ25 first needs to recognize and bind the siderophore receptor FhuA before it can be transported and delivered into cells (28), which suggests that MccY might as well. To evaluate the effect on the growth of *S.* Typhimurium under an iron-limiting condition, the growth of the parent strain (ST53) and the Δ*tonB*, Δ*exbB*, and Δ*exbD* mutants was determined in M63 medium (containing a low iron concentration) and iron-enriched M63 medium (50 μM FeSO_4_). In M63 medium, all the mutants exhibited an obvious delay (6 h) in growth initiation and decreased growth rates compared with that of the parent strain, and the maximum optical density (OD_max_; measured at 600 nm [OD_600_]) values of the Δ*tonB*, Δ*exbB*, and Δ*exbD* mutants were, respectively, 0.574 ± 0.015 (mean ± standard deviation), 0.589 ± 0.002, 0.577 ± 0.003, lower than that of the parent strain (0.767 ± 0.009) (*P < *0.0001) (Fig. 3A). The growth rates of the corresponding complementation strains were restored to levels similar to that of the parent strain in M63 medium. In the iron-enriched M63 medium, similar growth rates were observed for all mutants and the parent strain (Fig. 3B). Therefore, all of these results indicate that deletion of *tonB*, *exbB*, or *exbD* would adversely affect the growth of *S.* Typhimurium under iron-poor conditions.

Next, to further assess the effect of the MccY sensitivity in *S.* Typhimurium under a low-iron condition, we compared the MIC values of the Ton system mutants in M63 medium and iron-enriched M63 medium (Fig. 3C). In iron-enriched M63 medium, the MIC values of the wild-type and the mutants were 0.125 μM and 250 μM, respectively. In M63 medium, the MIC value of the wild-type strain was still 0.125 μM, while the MIC values of all the mutants decreased 2-fold, from 250 μM to 125 μM. Meanwhile, we evaluated the effect on the growth of *S.* Typhimurium when treated with 125 μM MccY under a low-iron condition or a high-iron condition. For the wild type treated with 125 μM MccY, the bacteria hardly grew under either the low-iron or the high-iron condition (Fig. 3D). However, the OD_max_ values of the Δ*tonB*, Δ*exbB*, and Δ*exbD* mutants treated with 125 μM MccY under the high-iron condition were 0.595 ± 0.028, 0.609 ± 0.012, and 0.567 ± 0.004, respectively, significantly higher than those of the Δ*tonB* (0.234 ± 0.043), Δ*exbB* (0.246 ± 0.018), and Δ*exbD* (0.256 ± 0.012) mutants treated with 125 μM MccY under the low-iron condition (*P < *0.001). These results indicated that the mutants were more sensitive to MccY under the low-iron condition.

Since the low-iron condition could increase the sensitivity of Ton system mutants to MccY, we further evaluated the effect on the siderophore production level of *S.* Typhimurium in the absence of the *tonB*, *exbB*, or *exbD* gene by chrome azurol S (CAS) assay. The parent strain exhibited 11.17- ± 0.41-mm yellow haloes, and the Δ*tonB* (14.83 ± 1.17 mm), Δ*exbB* (16.17 ± 0.41 mm), and Δ*exbD* (16.67 ± 0.82 mm) mutants showed significant increases in the yellow-orange halo diameter in the iron-limited environment (*P < *0.0001) (Fig. 3E). The siderophore production of all the complementation strains and overexpressed strains recovered to levels similar to that of the wild type. These results showed that the Ton system plays an important role in the iron utilization and siderophore production of *S.* Typhimurium and that the deletion of *tonB*, *exbB*, or *exbD* could increase the level of siderophore production.

In addition, to explore the competitive situation between siderophore and MccY for access to FhuA, an agar diffusion assay was performed for qualitative analysis. A concentration of 50 μg/mL of MccY was mixed with different concentrations of ferrichrome serially diluted from 1 mg/mL to 10^−4 ^mg/mL. The mixtures containing 50 μg/mL MccY and 10^−3^ or 10^−4 ^mg/mL ferrichrome produced clear inhibition zones consistent with that of the negative control (double-distilled water [ddH_2_O] instead of ferrichrome), whereas no inhibition zone was observed in the mixtures containing 50 μg/mL MccY and 10^−2^, 10^−1^, or 10^0^ mg/mL ferrichrome (Fig. 3F). These results suggested that a competition between ferrichrome and MccY to obtain FhuA can occur.

### Effects on physiological indicators of treatment of *S.* Typhimurium with MccY.

**FIG 4:**
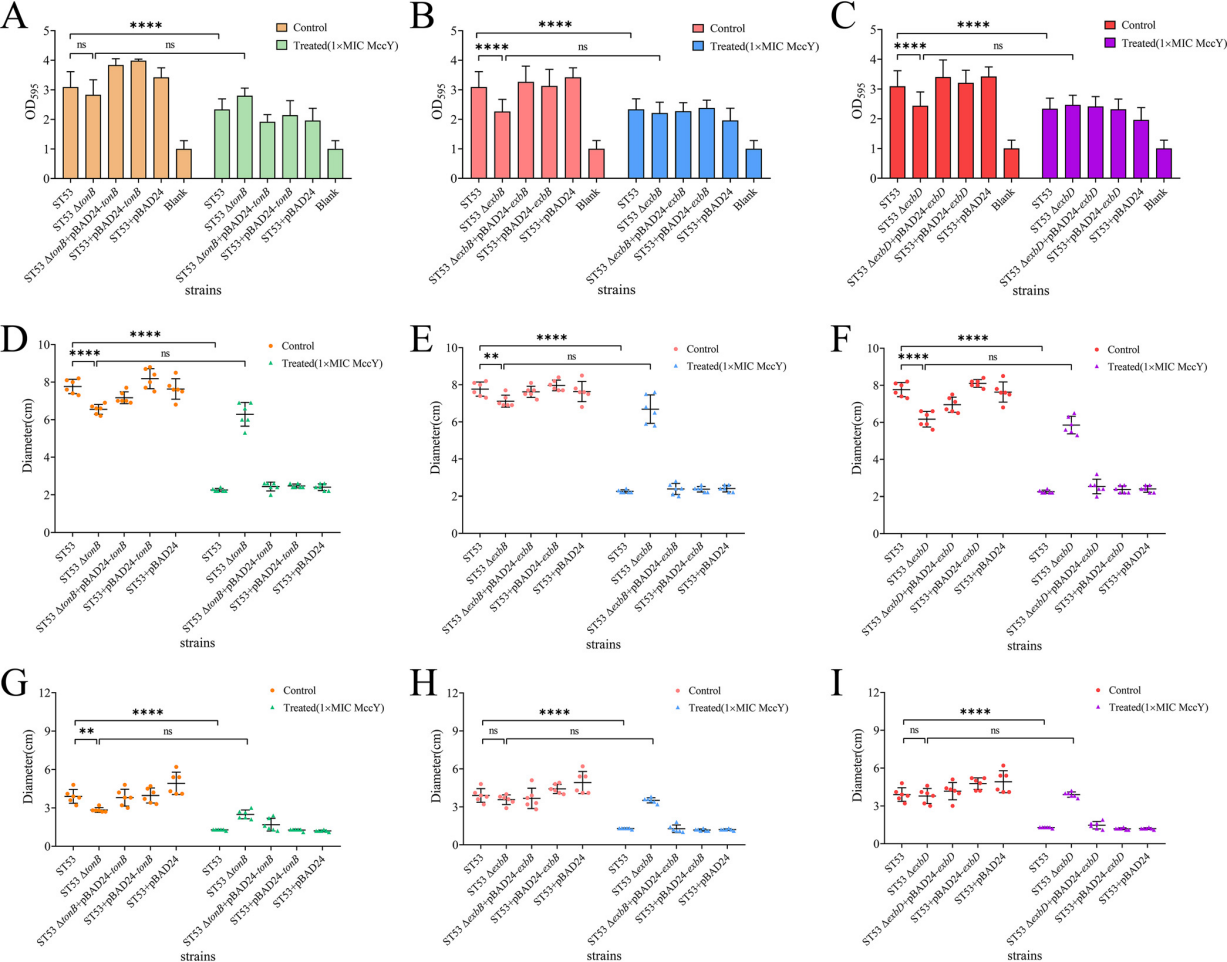
Impacts of the treatment with 1× MIC MccY or of *tonB*, *exbB*, or *exbD* deletion mutations on biofilm formation and swimming and swarming motilities in *S.* Typhimurium. (A, B, and C) The biofilm formation abilities of the strains were evaluated by the OD_595_ value. The blank spaces represent negative controls. (D, E, and F) The swimming motilities of these strains were analyzed on soft agar plates (0.3% agar) after 5 h of incubation at 37°C. (G, H, and I) Swarming motilities were analyzed on plates containing 0.5% agar after 10 h of incubation at 37°C. The OD_595_ values and the diameters of the swimming and swarming areas of wild-type and Ton system mutants treated with 1× MIC MccY were measured and compared to those of the control group without MccY, respectively, and the values of wild-type and Ton system mutants in the control group without MccY were also compared. Error bars show standard deviations. The level of significance was set at *P < *0.05. *, *P < *0.05; **, *P < *0.01; ***, *P < *0.001; ****, *P < *0.0001.

The swimming and swarming motilities and biofilm formation ability of Salmonella are related to its virulence (29–31). We further evaluated the effect of treatment with MccY and deletion of the *tonB*, *exbB*, or *exbD* gene on the virulence of *S.* Typhimurium through a series of microbiological experiments. With regard to biofilm formation ability, no obvious effects were observed in the Ton system mutants after the treatment with MccY at 1× MIC, while the parent strain treated with 1× MIC of MccY (OD_595_ = 2.34 ± 0.36) showed a significant decrease compared to the control (OD_595_ = 3.09 ± 0.52) (*P < *0.0001) (Fig. 4A to C). Furthermore, in the control group, the Δ*exbB* (OD_595_ = 2.26 ± 0.41) and Δ*exbD* (OD_595_ = 2.44 ± 0.46) mutants exhibited significant decreases compared with the biofilm formation ability of the parent strain (OD_595_ = 3.09 ± 0.52) (*P < *0.001). The recovery of the biofilm formation ability was obvious in all the complementation strains and the overexpressed strains. With regard to the swimming ability, the migration diameter of the parent strain after the treatment with 1× MIC MccY was 2.25 ± 0.08 cm, exhibiting a significant decrease compared with the migration diameter of the control (7.77 ± 0.38 cm) (*P < *0.0001), while none of the Ton system mutants showed obvious changes (Fig. 4D to F). In the control group, the migration diameters of the *tonB*, *exbB*, and *exbD* mutants were 6.55 ± 0.26 cm, 7.12 ± 0.33 cm, and 6.17 ± 0.42 cm, respectively, significantly smaller than that of the parent strain (7.77 ± 0.38 cm) (*P < *0.01). The swimming ability was restored in all complementation strains and overexpressed strains. With regard to the swarming ability, the migration diameter of the parent strain was 1.28 ± 0.04 cm after the treatment with 1× MIC MccY, significantly smaller than the migration diameter of the control (3.90 ± 0.54 cm) (*P < *0.0001), while each mutant with a mutation in the Ton system did not show any marked change (Fig. 4G to I). In the control group, the average migration diameter of the *tonB* mutant was 2.83 ± 0.19 cm, significantly smaller than that of the parent strain (3.90 ± 0.54 cm) (*P < *0.01). The recovery of swarming ability was obvious in all the complementation strains and overexpressed strains. All these findings showed that treatment with MccY or deletion of any gene in the Ton system could affect the biofilm formation ability and motility of *S.* Typhimurium and, thereby, might negatively affect the virulence of *S.* Typhimurium.

### Transcriptomic analyses of ST53 and the Δ*tonB*, Δ*exbB*, and Δ*exbD* mutants treated with MccY.

**FIG 5:**
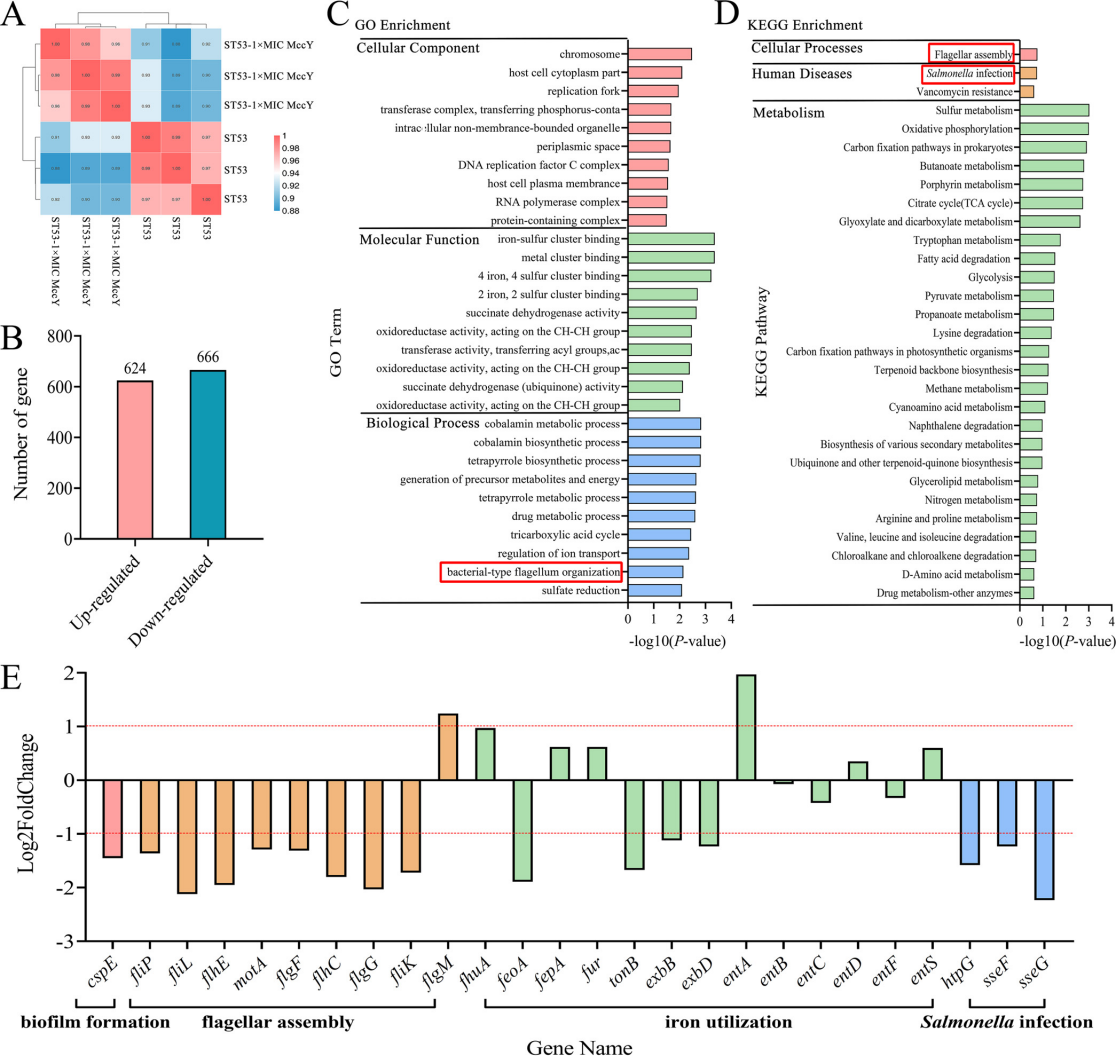
Transcriptomic analyses of ST53 treated with 1× MIC MccY. (A) Heatmap of gene expression patterns of ST53 and ST53 treated with 1×MIC MccY. The number in each square represents the correlation coefficient between samples. The closer the correlation coefficient is to 1, the higher the similarity of expression patterns between samples. (B) The number of DEGs in ST53 treated with 1× MIC MccY versus the gene expression in the ST53 group, including upregulated genes and downregulated genes. (C) GO terms for DEGs grouped into functional categories. GO classification was performed according to molecular function, biological process, and cellular component, and the top 10 GO term items with the smallest *P* values, that is, the most significant enrichment, were selected for display in each GO classification. (D) KEGG pathway classification enrichment of DEGs of MccY-treated ST53. *x* axis, the significance (−log *P* value) of KEGG pathway enrichment was calculated by the method of hypergeometric distribution; *y* axis, the categories of KEGG pathways. (D and E) Red boxes mark the pathways related to the physiological indicators of *S.* Typhimurium that we are interested in. (E) Expression levels of some important DEGs of 1× MIC MccY-treated ST53, involved in biofilm formation, flagellar assembly, iron utilization, and Salmonella infection. Horizontal red lines indicate a log_2_ fold change of +1 or −1.

To further evaluate the changes in transcription levels of genes in *S.* Typhimurium after exposure to 1× MIC MccY, we performed transcriptomic analyses (Tables S4 to S7 in supplemental file 2). Clustering heatmap analysis of 1× MIC MccY-treated strain ST53 (ST53-1× MIC MccY) and untreated ST53 showed that the gene expression patterns among the three samples from each condition were highly similar and had strong sample correlation (Fig. 5A). A comparison of 1× MIC MccY-treated and untreated ST53 showed that differential expression of 1,290 genes (≥2-fold change) was estimated by high-throughput RNA sequencing (RNA-seq), including 666 downregulated genes and 624 upregulated genes (Fig. 5B).

To further clarify the functions of differentially expressed genes (DEGs) in ST53-1× MIC MccY, we performed Gene Ontology (GO) and KEGG enrichment analysis of the DEGs (Fig. 5C and D). GO classification was performed according to molecular function (MF), biological process (BP), and cellular component (CC), and the top 10 GO term items with the smallest *P* values, that is, the most significant enrichment, were selected for display in each GO classification. The results showed that the DEGs encoded many proteins primarily related to chromosome (MF), iron-sulfur cluster binding (BP), and cobalamin metabolic process (CC). KEGG pathway enrichment clusters displayed the top 30 pathways with the smallest *P* values, that is, the most significant enrichment. The results revealed that genes primarily involved in cellular processes, diseases, and metabolism were significantly differentially expressed.

In the GO and KEGG classification of DEGs of ST53-1× MIC MccY, the proteins encoding the flagellar-assembly pathway, closely related to Salmonella motility, and the Salmonella infection pathway were also included (Fig. 5C and D). Considering that disturbance of MccY may have an impact on the virulence of *S.* Typhimurium, one DEG involved in biofilm, namely, *cspE* (downregulated), nine DEGs involved in flagellar assembly, namely, *fliK*, *fliP*, *fliL*, *flhE*, *motA*, *flgF*, *flhC*, and *flgG* (8 upregulated) and *flgM* (1 downregulated; negative regulator of flagellin synthesis), and three DEGs involved in Salmonella infection, namely, *htpG*, *sseF*, and *sseG* (downregulated), were selected to show the fold changes in these genes’ expression levels through RNA-seq results (Fig. 5E). Meanwhile, through analyzing transcriptomic results of 1× MIC MccY-treated Δ*tonB*, Δ*exbB*, and Δ*exbD* mutants, we found that the transcription levels of those genes, related to flagellar assembly, iron utilization, and Salmonella infection, had negligible changes in general, although some genes were upregulated (Fig. S3). These results at the transcript level further reflected that treatment with MccY may affect phenotypes like motility and even affect the infection ability of MccY-sensitive *S.* Typhimurium.

### Relating intramutant gene expression to gene function.

**FIG 6:**
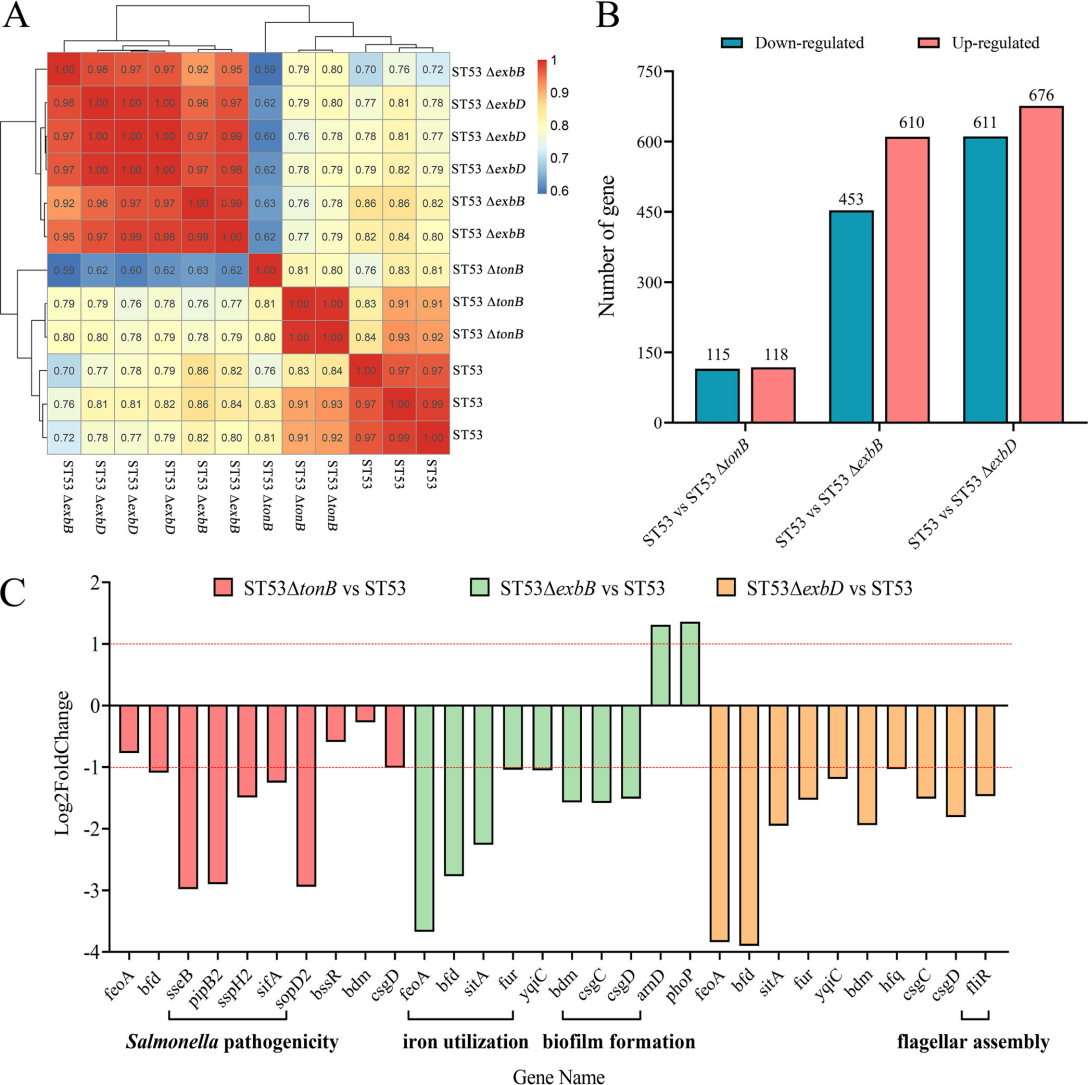
Transcriptomic analyses of *tonB*, *exbB*, and *exbD* deletion mutants. (A) The heatmap cluster of the sample correlation test shows the correlation of gene expression levels among different samples. The closer the correlation coefficient is to 1, the higher the similarity of expression patterns between samples. The different colors of the squares represent the correlation between the two samples. (B) The number of differentially expressed genes in each group, including upregulated genes and downregulated genes. (C) Expression levels of selected virulence genes in *S.* Typhimurium mutants relative to their expression in the wild type (ST53). The relative expression level for each gene is defined as the log_2_ fold change. Each color represents a comparison group composed of different gene deletion strains and the wild type. Horizontal red lines indicate a log_2_ fold change of +1 or −1.

To further evaluate the impact of *tonB*, *exbB*, or *exbD* gene deletion on the virulence of *S.* Typhimurium at the transcript level, we applied an RNA-seq method (Tables S8, S9, and S10 in supplemental file 3). The biological repetitions of each strain were basically clustered together by clustering heatmap analysis, indicating that the data were reproducible (Fig. 6A). Among them, a total of 233 differentially expressed genes (118 upregulated and 115 downregulated) were identified in ST53 Δ*tonB* versus the ST53 group (Fig. 6B), 1,063 differentially expressed genes (610 upregulated and 453 downregulated) were identified in ST53 Δ*exbB* versus the ST53 group, and 1,287 differentially expressed genes (676 upregulated and 611 downregulated) were identified in ST53 Δ*exbD* versus the ST53 group. Additionally, bacterial genes were assigned to functional groups to investigate the metabolic resources of every mutant. The functional categories of the significantly upregulated *S.* Typhimurium genes in the mutants showed that these genes were involved in protein transport, generation of precursor metabolites and energy, Salmonella infection, flagellar assembly, and so on (Fig. S4, S5, and S6).

Subsequently, we comprehensively analyzed the direct effect of *tonB*, *exbB*, or *exbD* gene deletion on *S.* Typhimurium virulence based on RNA-seq databases (Tables S8, S9, and S10). Consistent with the observed phenotypes of reduction in *S.* Typhimurium virulence by *tonB*, *exbB*, or *exbD* deletion, inactivation of these genes significantly inhibited the expression of several regulons known to mediate virulence in Salmonella (Fig. 6C), including the iron utilization-related *sitA*, *bfd*, *feoA*, and *fur* regulator genes, the *sseB*, *pipB2*, *sspH2*, *sifA*, *sopD2*, *hfq*, and *yqiC* (which alter the physiological functions of host cells and promote bacterial survival in host tissues) regulator genes associated with Salmonella pathogenicity, *bssR* and *bdm* (which is required to address changes in envelope pressure and environmental pressure), *csgC* (which plays a role in assembly into thin aggregated pilus fibers) and *csgD* (the main regulator of curly pili expression, which plays a positive role in biofilm formation) regulator genes associated with biofilm formation, and the flagellar-assembly-related *fliR* (which plays a role in flagellum biosynthesis) regulator gene. Moreover, CAS assay showed that the deletion of *tonB*, *exbB*, or *exbD* could increase the level of siderophore production, although the upregulation of genes related to siderophore synthesis was not observed in the transcriptome data (Fig. S7). Overall, the above-described results at the transcript level further reflected the idea that deletion of the *tonB*, *exbB*, or *exbD* gene may reduce the virulence of *S.* Typhimurium.

Finally, with *rpoD* as the internal reference gene (32), we randomly selected DEGs shared among the three comparison groups, namely, *pagC*, *sodC*, *zntA*, and *ackA*, for quantitative real-time PCR (qRT-PCR) to confirm the accuracy of the RNA-seq results. The results showed that the relative expression levels of the four DEGs were basically consistent with the results of RNA-seq (Fig. S8), indicating that the results of RNA-seq were credible and accurate.

## DISCUSSION

Salmonellosis is a significant foodborne disease, and animal-derived food is an important source of human infection with Salmonella disease, causing serious threats to human health. Antibiotics were originally the main method for treating salmonellosis; however, with the abuse of antibiotics in clinical treatment and livestock production, the drug resistance of Salmonella for various antibiotics, including beta-lactams, aminoglycosides, quinolones, sulfonamides, aminoglycosides, and tetracycline, is becoming increasingly serious (33). The severity of Salmonella drug resistance has aroused considerable interest in novel antimicrobials, such as microcins, that generally kill rapidly, have low-dose bactericidal biological activity, and are seldom reported to result in emerging bacterial resistance. MccY, a novel antibacterial agent with high specificity and efficacy against Salmonella infection, has recently been discovered and identified (19).

Microcins are a class of antimicrobial peptides that interact with cell surface receptors before translocation into susceptible bacteria (34). The antimicrobial activities of microcins are multifaceted, and they must cross both the outer membrane and the inner membrane and must find a cytoplasmic target (35). Our observations here showed that MccY is an RNA polymerase inhibitor *in vitro* (Fig. 1D). However, despite its high potency against its putative cytoplasmic target, MccY still has no activity against some bacteria tested previously (19). According to our previous study, MccY has strong structural similarities with MccJ25 but has a different antibacterial spectrum in various serotypes of Salmonella (19), and there is research reported showing that the outer membrane receptor FhuA is a determinant of susceptibility to MccJ25 (36). On this basis, we further explained the differences in the antibacterial spectra of MccY and MccJ25 in Salmonella strains in our previous study (19), which are caused by differences in FhuA originating from the two genotypes.

MccY is known to bind specifically to FhuA and SbmA, which must be essential for the ability of MccY to kill the target bacteria. Moreover, the above-described process requires an energy transport system (Ton or Tol-Pal system) to provide proton power to complete it (19). As expected, the production of MccY-resistant mutants confirmed that the proteins TonB, ExbB, and ExbD are required for the entry of MccY into target bacteria, which implies that the Ton system participates in the transport of MccY to the target strain. Moreover, TolQ and TolR in the Tol-Pal system are analogs of ExbB and ExbD (37, 38), and a previous study found that the function of ExbB in microcin L relaying on Ton system transfer could be replaced by its homolog TolQ (39). The *tolB*, *tolQ*, and *tolR* genes of the Tol-Pal system in *S.* Typhimurium were also knocked out, but these mutations did not affect the sensitivity of *S.* Typhimurium to MccY, indicating that the transport of MccY is independent of the Tol-Pal system.

Given a series of advantages of microcins, the view that drug resistance to microcins is unlikely to develop is generally accepted, because such a situation would involve changes in vital and highly conserved targets, which would necessarily be related to serious reductions in fitness and virulence (40). However, the contents of some previous articles published by our team (19) and others (21, 41) have challenged this view, showing that some bacteria, such as Salmonella and E. coli, can rapidly develop significant resistance to microcins. In our current study, deletion mutations of genes in the Ton system could confer resistance to MccY in *S.* Typhimurium. The MIC of the MccY-resistant mutants increased 2,000-fold compared with the MIC for the parent strain, and the mutants could survive high-concentration peptide treatment. As indicated by these results, it is obvious that a likely “Achilles’ heel” of MccY involves mutation in TonB, ExbB, or ExbD, which are located in the periplasmic membrane of susceptible cells. The Ton system is a multibasal membrane protein complex that is widely present in Gram-negative bacteria, and the proton gradient is used as an energy source to complete the transmembrane transport of substances (17). Thus, as with most antimicrobial compounds, mechanisms of resistance to microcins will inevitably emerge. Gene mutations in the Ton system might prevent the cellular uptake of MccY through the outer membrane, thereby preventing it from killing the target bacteria, resulting in Salmonella bacteria being highly resistant to MccY. In addition, a previous study demonstrated that the immune protein McjD confers MccJ25 immunity to host strains through efflux (42), and the presence of such proteins may also be an important cause of the resistance of pathogens to MccJ25-like lasso peptides. The MccY synthetic gene cluster contains the homologous gene *mcyD*, and the presence of the product of this gene may also lead to resistance of pathogens to MccY (19).

Free ferrous iron, ferric iron associated with chelators, and heme involved in bacterial growth and virulence are important metal iron sources. In animals, ferrous and ferric iron ions are present mainly in iron-binding proteins, resulting in low levels of free iron ions (43). For survival in iron-limited environments, most Gram-negative bacteria have evolved an iron uptake mechanism dependent on the Ton system (44). Here, we concluded that iron deficiency affected the growth of the mutants and improved the sensitivity of the mutants to MccY. We attempted to investigate why iron deficiency affected the susceptibility of *S.* Typhimurium to MccY. Studies have shown that bacteria usually secrete siderophores to capture iron ions in the environment, including iron-limited environments, as siderophores have the ability to specifically bind iron (45). Bacteria usually express specific outer membrane receptors to transport specific siderophores. The FhuA protein, as an important siderophore receptor in Salmonella, can chelate ferritin (46). We demonstrated that the mutants may secrete more siderophores to get iron for their own growth under iron restriction compared with the siderophore secretion level of the parent strain, and thus, the abundance of FhuA may increase under a low-iron condition. Our results also show a competitive relationship between ferrichrome and MccY for access to FhuA (Fig. 3F). Therefore, these observations in the laboratory led us to speculate that these strains may have captured more MccY through the siderophore receptor FhuA, which led to the deletion mutants being more sensitive to MccY under iron restriction, although the expression levels of *fhuA* had no obvious changes according to the RNA-seq data (Fig. S7).

At present, a key problem and concern is that drug resistance will be a real threat to the efficacy of MccY. Similar to antibiotics, it is necessary to carefully evaluate through experiments the potential risk of resistance to MccY for therapeutic purposes. Generally, mutations associated with antibiotic resistance are often accompanied by fitness costs, and growth rate, as a standard index, is used to measure the relative fitness of bacteria (47, 48). Surprisingly, compared with those of the parent strain, all the fitness parameters, including the growth rates that we observed, were changed in the deletion mutants, which might have a negative impact on the virulence of *S.* Typhimurium. Moreover, the effect of deletion of the *tonB*, *exbB*, or *exbD* gene on the expression levels of virulence-related genes of *S.* Typhimurium was consistent with the observed results for the fitness parameters. It is worth mentioning that in GO and KEGG analyses, the terms associated with differential gene enrichment clearly included the T3SS and Salmonella pathogenicity (Fig. S4, S5, and S6), which implied that deletion of the *tonB*, *exbB*, or *exbD* gene might have a great impact on the virulence of *S.* Typhimurium. In conclusion, according to our current understanding of gene transcription levels in the mutants, as determined by RNA-seq, and comprehensive phenotypic data of *S.* Typhimurium strains with Ton mutations, we believe that mutations in Ton system genes may occur under the selective pressure of MccY, which may perturb the function of this system and cause a series of physiological changes, including iron utilization and virulence reduction, in *S.* Typhimurium. Similarly, other studies also support our views, demonstrating that inactivation of *tonB* can have a strong negative effect on Salmonella virulence and on the exponential growth rate in laboratory medium (49, 50). The decrease of virulence in antimicrobial peptide-resistant strains is an interesting finding, since it suggests that resistant mutants might exhibit pathogenicity different from that of susceptible strains when growing in the host. Therefore, given the fitness costs, the potential preventive or therapeutic application of MccY should be judiciously considered.

### Conclusion.

Our study demonstrated that mutation in Ton system genes, including *tonB*, *exbB*, and *exbD*, could significantly increase MccY resistance in *S.* Typhimurium strains and that the sensitivity of Ton system mutants was increased under a low-iron condition. Our study also showed that treatment with MccY and deletion of Ton system genes reduced the virulence of *S.* Typhimurium, having a measurable negative impact on bacterial adaptability. Therefore, more in-depth studies of MccY should be performed and further developed to demonstrate its strong anti-Salmonella infection potential.

## MATERIALS AND METHODS

### Strains, plasmids, and growth conditions.

All the strains, plasmids, and primers used in this study are listed in Tables S1, S2, and S3. The *S.* Typhimurium parent strain, isolated from the laboratory, was used to generate mutants. All of the mutants, carrying a kanamycin box, were used for comparison with the parent strain in all experiments. We cloned the gene knockout constructs, including the *tonB*, *exbB*, and *exbD* genes, into the pMD19T vector. After amplification with the corresponding primers, we obtained pMD19T-*SttonB*, pMD19T-*StexbB*, and pMD19T-*StexbD*. All three plasmids were expressed from the pBAD24 vector. All the strains were grown at 37°C on Luria-Bertani liquid medium containing NaCl (10 g/L), yeast extract (5 g/L), and tryptone (10 g/L); Luria-Bertani solid medium had an additional supplement of 15 g/L agar. If necessary, M63 medium containing 1 M MgSO_4_·7H_2_O (1 mL/L), 20% glucose (10 mL/L), 0.5% vitamin B_1_ (0.1 mL/L), and 20% Casamino Acids (5 mL/L) or Mueller-Hinton (MH) broth (Oxoid; Thermo Fisher Scientific, USA) were used. Additionally, this study mainly used two antibiotics to make the resistance medium, kanamycin (30 μg/mL) or ampicillin (100 μg/mL) (both from Sigma-Aldrich).

### Construction of the *tonB*, *exbB*, or *exbD* mutant.

Construction of mutants was performed as described previously (51), with some modifications. The pKD46 plasmid with the λ-red system was transformed into *S.* Typhimurium ST53, and the homologous recombinant fragment was amplified from the pKD4 plasmid with tonB-mut-F and tonB-mut-R primers (Table S3), containing the kanamycin resistance box. Then, ST53 cells containing the pKD46 plasmid were prepared as competent cells. Finally, the homologous recombination fragment was transformed into the sensory cells by electroporation. The PCR products confirmed to be positive needed to be further verified by sequencing, which was carried out by Sangon Biotech, Shanghai, China. The knockout strain obtained was designated ST53 Δ*tonB*. The ST53 Δ*exbB*, ST53 Δ*exbD*, ST53 Δ*tolB*, ST53 Δ*tolQ*, and ST53 Δ*tolR* mutants were constructed by the same method with primers exbB-mut-F/R, exbD-mut-F/R, tolB-mut-F/R, tolQ-mut-F/R, and tolR-mut-F/R, respectively (Table S3).

### Cloning and complementation of the *tonB*, *exbB*, or *exbD* gene.

Construction of complementation strains was performed as described previously (52), with some modifications. The forward primer tonB-compl-F with the NdeI restriction enzyme site and the reverse primer tonB-compl-R containing the XbaI site were used for PCR amplification of *tonB*. The target fragment obtained was cloned into pBAD24 to obtain the recombinant plasmid pBAD24-*tonB*. After purification and sequencing, the plasmid was electroporated into *tonB* mutant cells to obtain a complementation strain that was named ST53 Δ*tonB*+pBAD24-*tonB*. Finally, positive monoclonal strains were selected and the expression of the pBAD24-*tonB* plasmid induced in the presence of 0.2% arabinose (Sangon Biotech, Shanghai). As mentioned above, the complementation strains ST53+pBAD24, ST53+pBAD24-*tonB*, ST53+pBAD24-*exbB*, ST53+pBAD24-*exbD*, ST53 Δ*exbB*+pBAD24-*exbB*, and ST53 Δ*exbD*+pBAD24-*exbD* were constructed by the same method.

### Preparation and quantification of MccY.

The methods used for MccY expression, purification, and quantification were performed as described previously (19). The expression of MccY was induced by culture for 6 h in M9 medium to which 30 μg/mL kanamycin and 1 mM IPTG (isopropyl β-d-thiogalactopyranoside) were added. Then, the supernatant was collected with high-speed centrifugation at 4°C. Afterward, as previously described (19), MccY from the harvested supernatant was enriched by reversed-phase solid-phase extraction (SPE) and then purified and quantified by high-performance liquid chromatography (HPLC). Finally, MccY was quantified at 1.5 mg/mL for the test.

### Antimicrobial activity test.

As described in previous studies, MccY inhibition tests were performed on solid medium (53). Specifically, the strains were resuscitated on LB agar plates, and single colonies were picked out into 1 mL LB broth the next day and cultured to an OD_600_ of 0.8 at 37°C and 200 rpm. Then, the bacterial liquid (1:1,000) was added to LB medium cooled to 40°C and supplemented with 0.5% agar to prepare growth plates. Next, MccY was serially diluted from 250 μM to 0.125 μM, and 50-μL amounts of different concentrations of MccY were placed in the centers of the soft agar plates and cultured at 37°C for 1 day to observe whether a bacteriostatic zone was formed on the surface of the plate to evaluate the activity.

### RNAP inhibition assay using MccY in various concentrations.

RNAP inhibition was evaluated *in vitro* using quantitative PCR (qPCR). MccY was used in triplicate at concentrations of 5, 1, 0.1, 0.01, 0.001, and 0.0001 μM. Briefly, each 50-μL reaction mixture contained 35.5 μL nuclease-free water or MccY solution, 10 μL RNAP reaction buffer, 1 μL ribonucleotide solution mix, 1 μL E. coli RNAP (NEB, USA), 0.5 μL RNase inhibitor (Sangon Biotech, Shanghai), and 2 μL (500 ng) MG1655 genome DNA. The reaction mixtures were incubated overnight at 37°C to synthesize standard RNA; the same reaction conditions without E. coli RNAP were defined as the negative control. Subsequently, a bacterial RNA kit (Fastagen, Shanghai) and StarScript III RT mix with gDNA remover kit (GenStar, Shanghai) were used to extract the total RNA and perform reverse transcription. Finally, qPCR was used to determine the transcriptional level of the *rpsM* gene (in E. coli K12 strain MG1655) in each reaction system. The transcriptional level of the *rpsM* gene in the positive-control group (containing E. coli RNAP without MccY) served as the baseline to calculate the fold changes in the transcriptional levels of the *rpsM* gene in the other groups.

### MccY susceptibility assays.

MIC and killing curves were performed with 96-well microplates. Strains were resuscitated on MH plates, and single colonies were taken the next day and cultured in MH broth at 37°C and 200 rpm until reaching an OD_600_ of 0.8. After dilution to 1:1,000, 50-μL amounts were added into 96-well plates. Then, MccY with an initial concentration of 250 μM was continuously diluted to 0.0125 μM in 2-fold and 5-fold gradients, 50-μL amounts of diluted MccY of different concentrations were added into 50-μL amounts of diluted bacteria successively, and the mixtures were cultured at 37°C for 24 h to define the MIC values by measuring the *A*_600_. Assays for killing curves were performed through coincubation and cell counts. Concentrations of 0, 0.125, 1.25, 12.5, and 125 μM MccY were added to 50-μL amounts of bacterial solution cultured to 5 × 10^6^ CFU/mL, and the mixtures incubated for 24 h at 37°C. After 0, 0.5, 4, and 24 h, samples were diluted and cell counts performed. Then, the ratios of the numbers of living cells in the samples treated with MccY and the numbers of living cells in the blank control group were calculated and multiplied by 100 to obtain the bacterial survival rate at each time point, so as to draw the killing curve.

### Growth kinetics.

The wild-type ST53, the *tonB*, *exbB*, and *exbD* mutants, and the respective complementation strains were resuscitated on LB plates. Then, all strains were subjected to growth rate assays in M63 medium (containing a low iron concentration) and iron-enriched M63 medium (50 μM FeSO_4_). When necessary, the cultures were treated with 125 μM MccY. Specifically, the recovered strains were cultured in each of the above-described broths until reaching an OD_600_ of 1. This suspension was then diluted again to a final OD_600_ of 0.1 in fresh broth and aliquoted at 350 μL per well into 100-well plates. Subsequently, the BioScreen C (Bioscreen; Oy Growth Curves AB Ltd.) was used to automatically measure the OD_600_ of each well in the plate. The culture conditions were set as 37°C and 200 rpm, and the reading intervals were every 2 h for a total of 72 h. The negative-control groups were not inoculated with bacteria. The experiments were repeated three times. Finally, growth curves were generated using the recorded data.

### CAS assays.

Chrome azurol S (CAS) is a blue medium containing iron ions, and when high-iron-affinity chelators like siderophores take away the iron, it forms yellow-orange haloes around growing bacterial colonies, which can be used to detect the endogenous siderophores of Salmonella bacteria (54). The strains were cultured at 37°C and 200 rpm overnight in low-iron LB medium treated with dipyridine. After collecting bacteria the next day by centrifugation and washing 3 times with phosphate-buffered saline (PBS), the bacteria were diluted with PBS to an OD_600_ of 0.1. Then, 10-μL amounts were placed in the centers of the CAS agar plates and cultured for 18 h at 37°C. Finally, the amounts of siderophores were evaluated via the diameters of the yellow-orange haloes around the colonies. The experiments were repeated three times.

### Competition assays between siderophores and MccY.

The ST53 strain was resuscitated on an LB agar plate, and the next day, a single colony was selected into 1 mL LB broth and cultured at 37°C and 200 rpm until the OD_600_ was 0.8. The bacterial liquid (1:1,000) was added to LB medium cooled to 40°C and supplemented with 0.5% agar to prepare growth plates. Ferrichrome was then serially diluted from 1 mg/mL to 10^−4 ^mg/mL. The mixtures containing 5-μL amounts of various concentrations of ferrichrome and 5 μL of 50 μg/mL MccY were placed at the center of each area of the soft plates (the mixture without ferrichrome was used as a negative control) and cultured at 37°C for 24 h to determine if a bacteriostatic zone formed on the surface of the plate.

### Determination of biofilm formation by crystal violet staining.

As described in previous studies (55), crystal violet staining was used to quantify the biofilms of bacterial strains, with some modifications. From resuscitated strains on LB agar plates, single colonies were added to fresh LB broth the next day and cultured until reaching an OD_600_ of 0.8. When necessary, the cultures were challenged with 0.125 μM (1× MIC) MccY for 1 h. Then, salt-free LB broth was used to dilute the cultures at a ratio of 1:100, and 200-μL amounts of the diluted bacteria were inoculated into 96-well plates. The blank control was a salt-free LB broth without bacteria. After incubating at 27°C for 3 days, the culture from each well was carefully discarded, and 200 μL of anhydrous methanol added for 15 min, fixing the samples. Then, anhydrous methanol was gently discarded from each well, the plates dried, and 200 μL of 1% crystal violet solution added, staining for 15 min. Then, the unbound dye was slowly removed and 200 μL of PBS added to each well for washing, repeating three times. Finally, 200 μL of 33% acetic acid was added to each well, thereby dissolving the crystal violet, and the OD_595_ was determined with a microplate reader. The above-described operation required three biological repetitions for each sample and control, and the experiments were repeated three times.

### Swimming and swarming capabilities.

To detect the motility of the bacteria, 10 μL of bacterial solution cultured overnight until reaching an OD_600_ of 0.8 (when necessary, overnight cultures were challenged with 0.125 μM [1× MIC] MccY for 1 h) was spotted gently in the middle of swarm plates (10 g tryptone, 5 g yeast powder, 5 g NaCl, and 5 g agar powder added to 975 mL ddH_2_O and 25 mL of 20% glucose) or swim plates (10 g tryptone, 5 g yeast powder, 5 g NaCl, and 3 g agar powder added to 1 L ddH_2_O) and cultured at 37°C for 10 h and 5 h, respectively. The rates of migration of the bacteria from the point of inoculation (observed as a turbid zone, measured in centimeters) were measured according to Kim and Surette (56). The experiments were repeated three times.

### Total RNA extraction, library preparation, and deep sequencing.

Total RNA extraction, cDNA library preparation, and Illumina sequencing were performed by Nanjing Personal Gene Technology Co., Ltd. (China). Total RNA was obtained from Salmonella samples (including 3 biological replicates of ST53 and its Δ*tonB*, Δ*exbB*, and Δ*exbD* mutants and of ST53 and the Δ*tonB*, Δ*exbB*, and Δ*exbD* mutants treated with 0.125 μM MccY for 1 h) by using a bacterial RNA kit according to the manufacturer’s instructions, followed by detection of total RNA concentration and purity. Then, the rRNA was removed. The RNA samples were fragmented to 200 to 300 bp by ion interruption. Synthesis of the first strand of cDNA was accomplished by the action of random primers and reverse transcriptase. A specific library was built, and dTTP was replaced by dUTP to synthesize the second strand of cDNA.

Then, library fragments were enriched by electrophoresis, and library size was measured using an Agilent 2100 Bioanalyzer. The cDNA libraries were sequenced on an Illumina HiSeq 2000 system. The expression levels of each gene in the sample were calculated by sequence alignment using the Salmonella reference genome. Subsequently, the samples were divided into seven groups, namely, ST53-1× MIC MccY versus ST53, ST53 Δ*tonB*-1× MIC MccY versus ST53 Δ*tonB*, ST53 Δ*exbB*-1× MIC MccY versus ST53 Δ*exbB*, ST53 Δ*exbD*-1× MIC MccY versus ST53 Δ*exbD*, ST53 Δ*tonB* versus ST53, ST53 Δ*exbB* versus ST53, and ST53 Δ*exbD* versus ST53; further expression difference analysis, enrichment analysis, and cluster analysis were performed for these groups. All raw data were submitted to the GEO database (accession number GSE214184).

### RNA extraction and qRT-PCR.

The strains of *S.* Typhimurium were grown in LB broth until reaching an OD_600_ of 0.6 to 0.8, and a bacterial RNA kit (Omega) was used to extract total RNA. Additionally, the PrimeScript RT reagent kit with gDNA Eraser (TaKaRa, Beijing) was used for reverse transcription. Quantitative real-time PCR (qRT-PCR) was performed using Hieff qPCR SYBR green master mix (no ROX) (Yeasen) in a fluorescence-detecting quantitative PCR instrument (Bio-Rad). Finally, we used *rpoD* as the reference gene (32), and relative changes in mRNA levels were analyzed according to Pfaffl (57).

### Statistical data analysis.

The *t* test was used for statistical analyses, setting the level of significance as a *P* value of <0.05. GraphPad Prism version 8.0 was used for analysis.

### Data availability.

RNA-seq data reported in this paper are deposited in the NCBI Gene Expression Omnibus with accession number GSE214184. The complete data set is available in supplemental files 2 and 3. All other relevant data are available from the corresponding author upon request.
