# Supplementary material for: Resistance Mechanism and Physiological Effects of Microcin Y in Salmonella enterica subsp. enterica Serovar Typhimurium
Source: Microbiol Spectr. 2022 Dec 1;10(6):e01859-22. doi: 10.1128/spectrum.01859-22 (PMC9769762; doi:10.1128/spectrum.01859-22)
Supplement: Supplemental file 1 — Fig. S1 to S8 and Tables S1 to S3. Download spectrum.01859-22-s0001.pdf, PDF file, 2.8 MB [file spectrum.01859-22-s0001.pdf]

Supplementary Material

The Resistance Mechanism and Physiological Effects of Microcin Y in *Salmonella enterica* subsp. *enterica* serovar Typhimurium

Authors: Yu Han[a], Yu Li[a], Zhiwei Zeng[a], Wenjing Li[a], Saixiang Feng[a,b,c,d,e]*, Weisheng Cao[a,b,c,d,e]*

[a]College of Veterinary Medicine, South China Agricultural University, Guangzhou, China.

[b]Key Laboratory of Zoonosis Prevention and Control of Guangdong Province, China.

[c]Key Laboratory of Zoonosis of Ministry of Agriculture and Rural Affairs, Guangzhou, China.

[d]Key Laboratory of Veterinary Vaccine Innovation of the Ministry of Agriculture and Rural Affairs, Guangzhou, China.

[e]National and Regional Joint Engineering Laboratory for Medicament of Zoonosis Prevention and Control, Guangzhou, China.

***Correspondence:**

Saixiang Feng: Tel/Fax, +86-20-85280718; E-mail: fengsx@scau.edu.cn

Weisheng Cao: Tel/Fax, +86-20-85282536; E-mail: caoweish@scau.edu.cn

Supplementary Figure S1

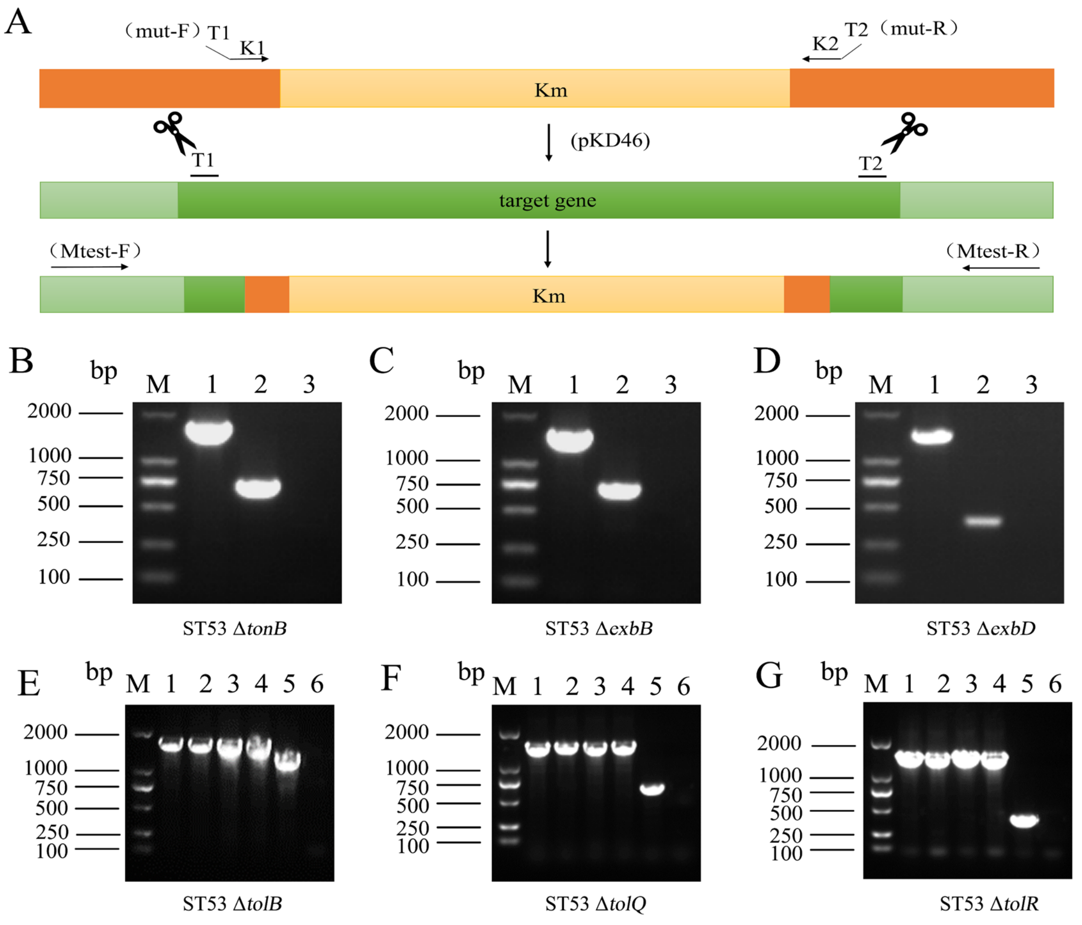

Figure S1. Construction of gene mutants. (A) Schematic diagram of the construction process of gene mutants. The upstream primer T1of the target gene and the upstream primer K1 of Km form the F end of the homology arm, namely, mut-F, and the upstream primer T2 of the target gene and the upstream primer K2 of Km form the R end of the homology arm, namely, mut-R. Amplify the complete Km cassette using forward primer mut-F of the homology arm and reverse primer mut-R of the homology arm. Through the λ-Red system to perform homologous recombination, and the Km box was inserted into the target gene to complete the construction of mutant strain. The primer outside the target genes (Mtest-F/R) were used to verify the mutants. The 24 primers used in the study are listed in Table S3. (B) PCR to confirm *tonB* mutant with the

primers tonB-Mtest-F/R. Lane M, DNA molecular marker; lane 1, ST53 Δ*tonB*; lane 2, ST53; lane 3, negative control (ddH_2O as template). (C) PCR to confirm *exbB* mutant with the primers exbB-Mtest-F/R. Lane M, DNA molecular marker; lane 1, ST53 Δ*exbB*; lane 2, ST53; lane 3, negative control (ddH_2O as template). (D) PCR to confirm *exbD* mutant with the primers exbD-Mtest-F/R. Lane M, DNA molecular marker; lane 1, ST53 Δ*exbD*; lane 2, ST53; lane 3, negative control (ddH_2O as template). (E) PCR to confirm *tolB* mutant with the primers tolB-Mtest-F/R. Lane M, DNA molecular marker; lanes 1, 2, 3 and 4, ST53 Δ*tolB*; lane 5, ST53; lane 6, negative control (ddH_2O as template). (F) PCR to confirm *tolQ* mutant with the primers tolQ-Mtest-F/R. Lane M, DNA molecular marker; lanes 1, 2, 3 and 4, ST53 Δ*tolQ*; lane 5, ST53; lane 6, negative control (ddH_2O as template). (G) PCR to confirm knockout of *tolR* mutant with the primers tolR-Mtest-F/R. Lane M, DNA molecular marker; lanes 1, 2, 3 and 4, ST53 Δ*tolR*; lane 5, ST53; lane 6, negative control (ddH_2O as template).

Supplementary Figure S2

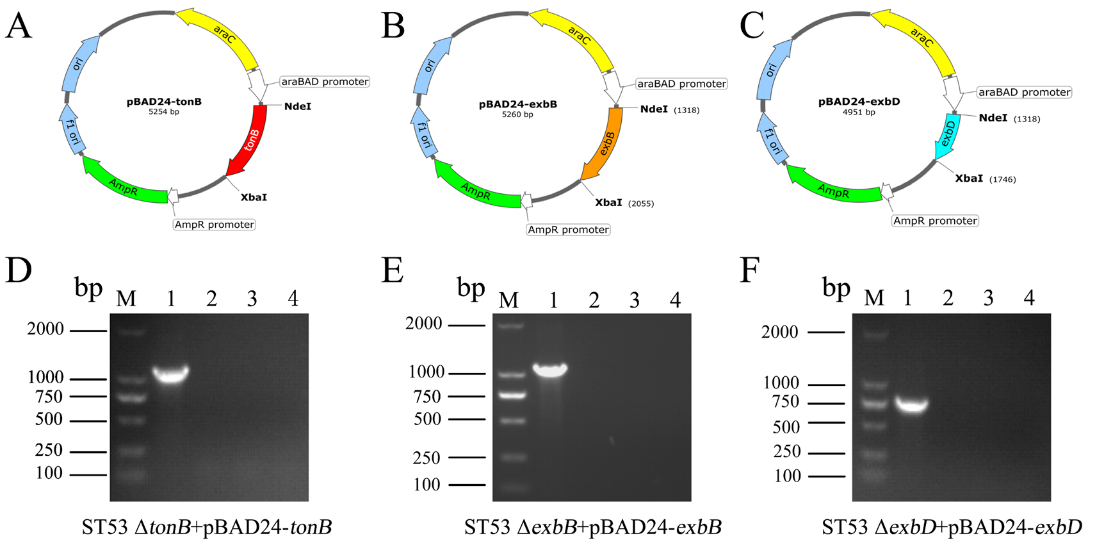

Figure S2. Construction of *tonB*, *exbB* or *exbD* gene complementary strain. Maps of recombinant expression plasmids. The target gene cluster was PCR-amplified and cloned between the *Nde* I and *Xba* I enzyme sites. The different colors indicate various genes, including *tonB*, *exbB* and *exbD* genes. The arrows indicate the positions and directions of transcription. The circular maps were generated using SnapGene software (from GSL Biotech; available at snapgene.com). (A) Recombinant expression plasmid: pBAD24-tonB. (B) Recombinant expression plasmid: pBAD24-exbB. (C) Recombinant expression plasmid: pBAD24-exbD. The constructed recombinant expression plasmids were electroporated into the corresponding deletion mutants to obtain the respective complementary strains. The 12 primers used in the study are listed in Table S3. (D) PCR to confirm pBAD24-*tonB* in ST53 Δ*tonB*+pBAD24-*tonB* with the primers pBAD24-F/R. Lane M, DNA molecular marker, DS2000; lane 1, ST53 Δ*tonB*+pBAD24-*tonB*; lane 2, ST53 Δ*tonB*; lane 3, ST53; lane 4, negative control (ddH_2O as template). (E) PCR to confirm pBAD24-*exbB* in ST53 Δ*exbB*+pBAD24-

exbB with the primers pBAD24-F/R. Lane M, DNA molecular marker, DS2000; lane 1, ST53 Δ*exbB*+pBAD24-*exbB*; lane 2, ST53 Δ*exbB*; lane 3, ST53; lane 4, negative control (ddH_2O as template). (F) PCR to confirm pBAD24-*exbD* in ST53 Δ*exbD*+pBAD24-*exbD* with the primers pBAD24-F/R. Lane M, DNA molecular marker, DS2000; lane 1, ST53 Δ*exbD*+pBAD24-*exbD*; lane 2, ST53 Δ*exbD*; lane 3, ST53; lane 4, negative control (ddH_2O as template).

Supplementary Figure S3

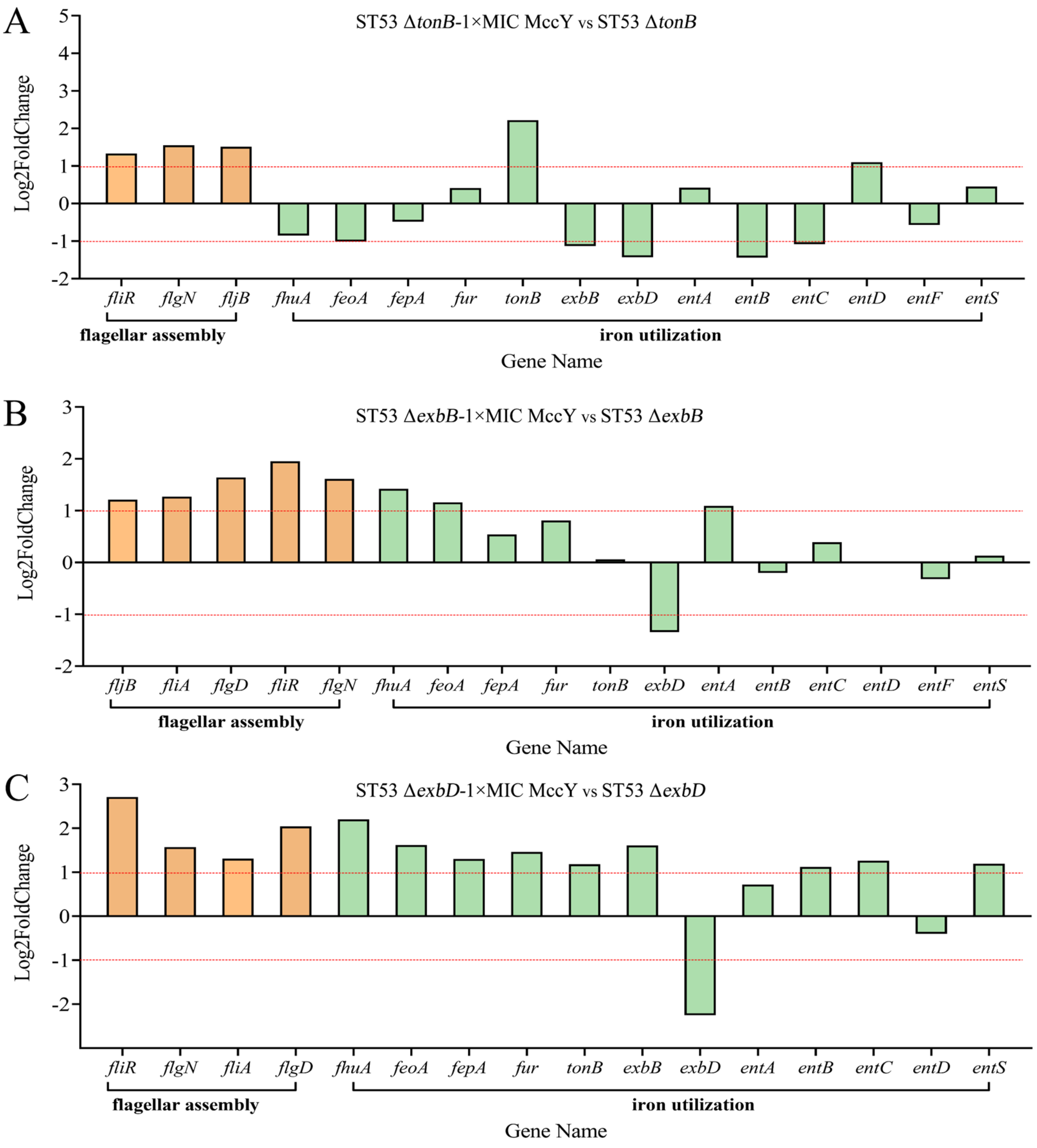

Figure S3. Expression levels of important DEGs in each group treated with 1× MIC MccY, involved in flagellar assembly and iron utilization. (A) ST53 Δ*tonB*-1×MIC MccY vs ST53 Δ*tonB* group, (B) ST53 Δ*exbB*-1×MIC MccY vs ST53 Δ*exbB* group (C) ST53 Δ*exbD*-1×MIC MccY vs ST53Δ*exbD* group. The red curve: Log2 fold change=1.

Supplementary Figure S4

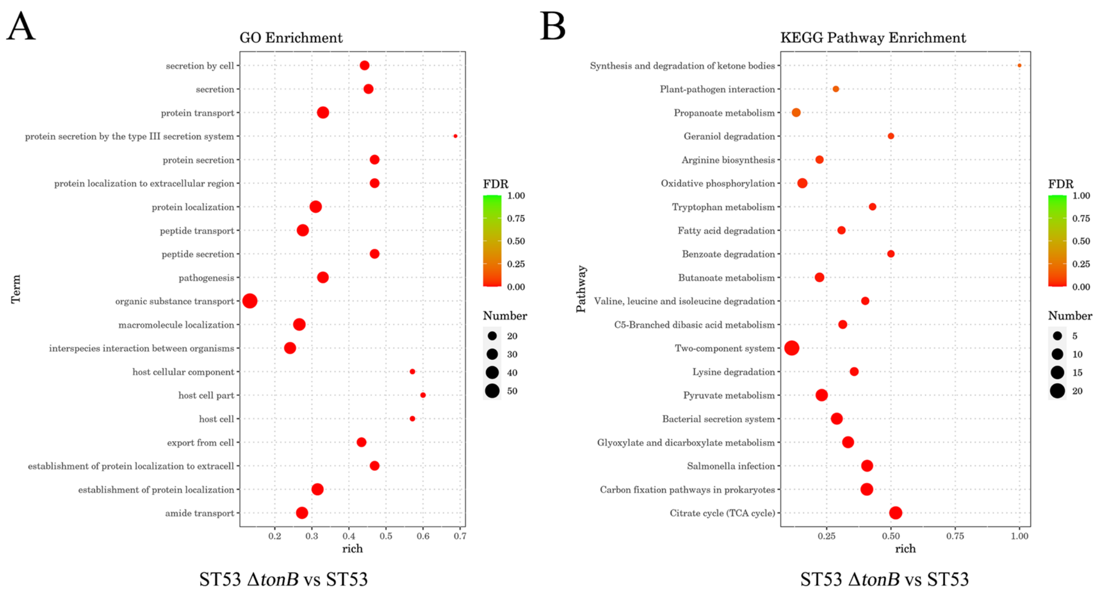

Figure S4. GO and KEGG analysis of ST53 Δ*tonB* vs ST53 group in RNA-seq. The functions of genes were classified according to the GO and KEGG databases and genomic pathway database, and the top 20 items with the smallest p value, i.e., the most significant enrichment, were selected for display. The degree of enrichment was measured by rich factor, FDR value and the number of genes enriched in this pathway. (A) Bubble diagram of GO enrichment analysis in ST53 Δ*tonB* vs ST53 group. (B) Bubble diagram of KEGG enrichment analysis in ST53 Δ*tonB* vs ST53 group. Rich factor refers to the ratio of the number of differentially expressed genes enriched in the pathway to the number of differentially expressed genes annotated. The larger the rich factor is, the greater the degree of enrichment. The general value range of FDR is 0-1. The closer it is to zero, the more significant the enrichment is.

Supplementary Figure S5

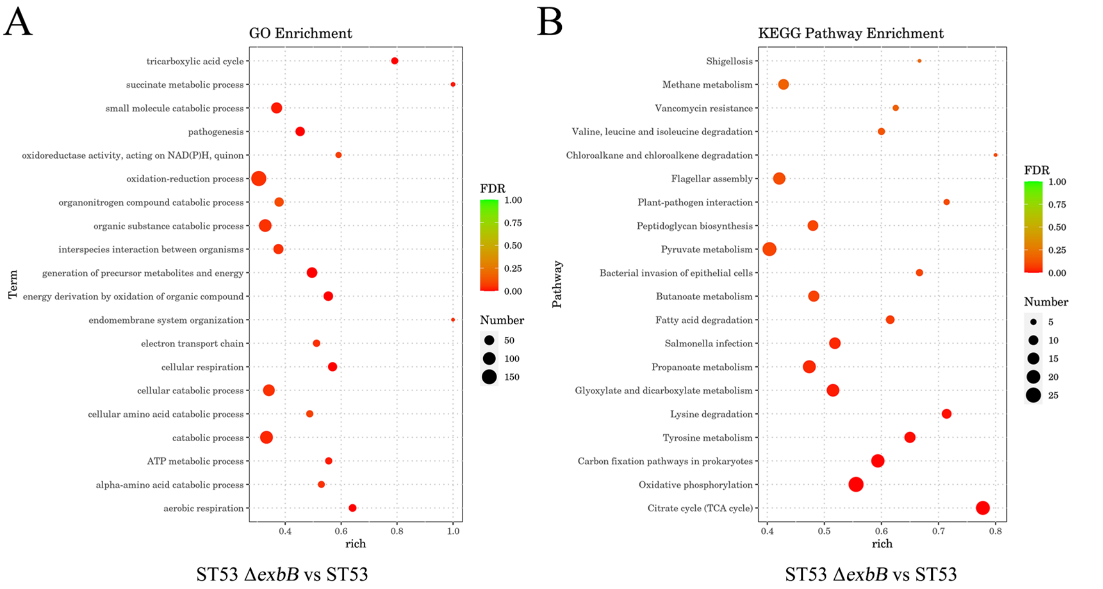

Figure S5. GO and KEGG analysis of ST53 Δ*exbB* vs ST53 group in RNA-seq. (A) Bubble diagram of GO enrichment analysis in ST53 Δ*exbB* vs ST53 group. (B) Bubble diagram of KEGG enrichment analysis in ST53 Δ*exbB* vs ST53 group.

Supplementary Figure S6

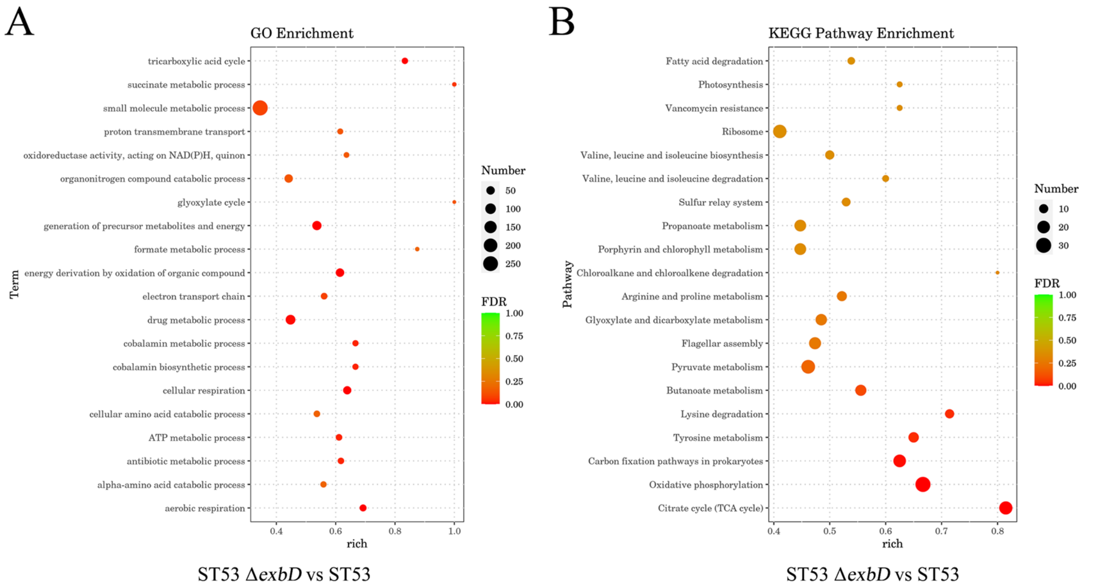

Figure S6. GO and KEGG analysis of ST53 Δ*exbD* vs ST53 group in RNA-seq. (A) Bubble diagram of GO enrichment analysis in ST53 Δ*exbD* vs ST53 group. (B) Bubble diagram of KEGG enrichment analysis in ST53 Δ*exbD* vs ST53 group.

Supplementary Figure S7

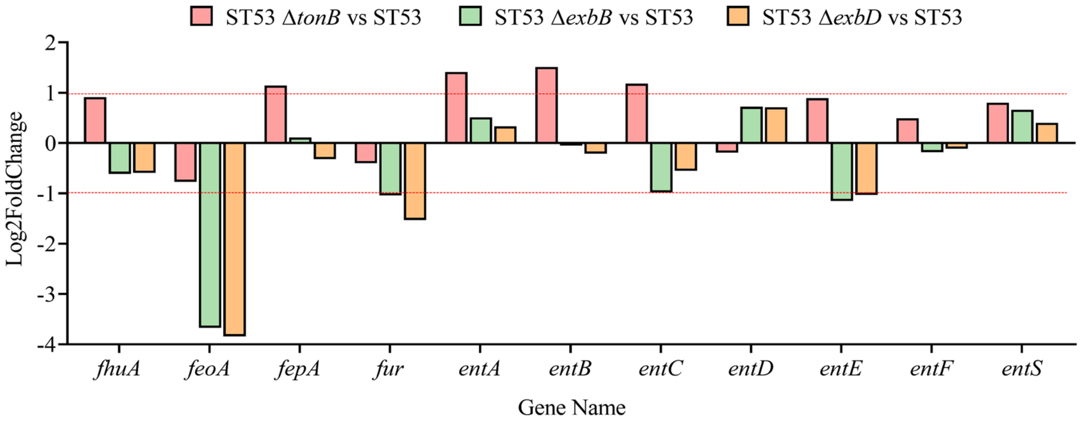

Figure S7. Expression levels of some important DEGs in each group, involved in siderophore utilization and production. Red represents ST53 Δ*tonB* vs ST53 group, green represents ST53 Δ*exbB* vs ST53 group, orange represents ST53 Δ*exbD* vs ST53 group. The red curve: Log2 fold change=1.

Supplementary Figure S8

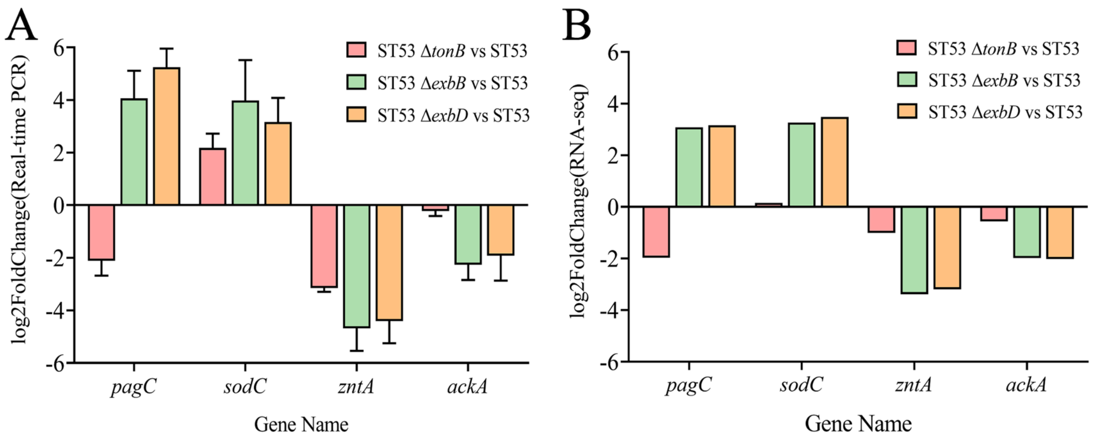

Figure S8. Verification of RNA-seq accuracy with qRT–PCR. Four genes were randomly selected to compare their relative expression in RNA-seq and qRT–PCR. (A) Relative expression results for each group measured by qRT–PCR. (B) Relative expression results for each group measured by RNA-seq.

Supplementary Table S1

Table S1. Strains used in this study.

Name	Relevant characteristic(s)	Source
ST53	*Salmonella* Typhimurium isolate, wild type	Laboratory collection
DH5α	F^-, φ80d/*lacZ*ΔM15, Δ(*lacZYA-argF*) U169 *recA*1 *endA*1 *hsdR*17	Laboratory collection
BL21	F^-, *ompT hsdS$_B$* (r_B^-, m_B^-) *gal dcm* (DE3)	Laboratory collection
ST53 Δ*tonB*	*tonB* inactivation mutant of *S.* Typhi	This study
ST53 Δ*exbB*	*exbB* inactivation mutant of *S.* Typhi	This study
ST53 Δ*exbD*	*exbD* inactivation mutant of *S.* Typhi	This study
ST53 Δ*tolB*	*tolB* inactivation mutant of *S.* Typhi	This study
ST53 Δ*tolQ*	*tolQ* inactivation mutant of *S.* Typhi	This study
ST53 Δ*tolR*	*tolR* inactivation mutant of *S.* Typhi	This study
ST53 Δ*tonB*+pBAD24-*tonB*	*tonB* complementation strain of *S.* Typhi	This study
ST53 Δ*exbB*+pBAD24-*exbB*	*exbB* complementation strain of *S.* Typhi	This study
ST53 Δ*exbD*+pBAD24-*exbD*	*exbD* complementation strain of *S.* Typhi	This study

Supplementary Table S2

Table S2. Plasmids used in this study.

Name	Relevant characteristic(s)	Source
pKD46	*E. coli* lambda Red recombineering expression vector	Laboratory collection
pKD4	Donor of kanamycin resistance cassette	Laboratory collection
pMD19T	Cloning vector, Amp^R	Laboratory collection
pMD19T-*SttonB*	*Salmonella* Typhimurium *tonB* gene cloned in pMD19T, Amp^R	This study
pMD19T-*StexbB*	*Salmonella* Typhimurium *exbB* gene cloned in pMD19T, Amp^R	This study
pMD19T-*StexbD*	*Salmonella* Typhimurium *exbD* gene cloned in pMD19T, Amp^R	This study
pBAD24	Expression vector, Amp^R	Laboratory collection
pBAD24-*SttonB*	*Salmonella* Typhimurium *tonB* gene cloned in pBAD24 with *Nde* I and *Xba* I, Amp^R	This study
pBAD24-*StexbB*	*Salmonella* Typhimurium *exbB* gene cloned in pBAD24 with *Nde* I and *Xba* I, Amp^R	This study
pBAD24-*StexbD*	*Salmonella* Typhimurium *exbD* gene cloned in pBAD24 with *Nde* I and *Xba* I, Amp^R	This study

Supplementary Table S3

Table S3. Primers used in this study.

Primer	Sequence (5' to 3')
pKD46-F	GCAACTTTATCCGCCTCC
pKD46-R	TCGCCCTTATTCCCTTTT
pKD4-F	GCATGATTGAACAAGATGGATTGC
pKD4-R	CGCTCAGAAGAACTCGTCAAGAA
tonB-mut-F	CTGGCCGACACTCCTTTCCGTAGGCATTCATGGTGCTGTCGTCTTGAGCGATTGTGTAG
tonB-mut-R	AGATAATATTGACCACCAGCCCGGAACCCGGCTTGCCTGAATTAGCCATGGTCCATATG
exbB-mut-F	CAGCACGCCGATATTGTCGTGAAGTGCGTGATGATTGGTTTGATTCTGGCGTCAGTCGT CACCTGGGCTTGCTAAAGGAAGCGGAACAC
exbB-mut-R	TACCGGCTGCGCACTGGCGCTCGCATTCAGATCAAGATCGCGGCTTTGCAGCAGCAATA CCTGCGCCGCGAAGAACTCCAGCATGAGA
exbD-mut-F	GACAACGGCGAAATGCATGAAATCAACGTGACGCCGTTTATCGATGTCATGTTGGTTCT GCTGATTATCTGCTAAAGGAAGCGGAACAC
exbD-mut-R	GACCAGACCAATCTTGAGATAGCCCGCCTGATGCAGCATATCCATCACTTTCATCAGGG TTTCATACTCCGAAGAACTCCAGCATGAGA
tonB-Mtest-F	CTTGATTTACCTCGTCGCTT
tonB-Mtest-R	AATCTGCGCCGTACCGTTCA
exbB-Mtest-F	GATGCAGACGGATCTTTCCG
exbB-Mtest-R	CCCTACCCGTAATTTCTGCG
exbD-Mtest-F	CGTCTTAACGAGAACCTGGA
exbD-Mtest-R	TTTCGCTTTCGCGGTCTCTT
tolB-mut-F	TTTGGTTTTCTGATGCTGTGGGCGGCGGTGCTGCACGCACGTCTTGAGCGATTGTGTAG
tolB-mut-R	GCGATTTCACCTGACCATCAGTTGCCGGAAGACGCGCTTAATTAGCCATGGTCCATATG
tolQ-mut-F	GATTTTGATTGGTTTTTCAATAGCATCCTGGGCCATCATTATCCAGCGAACGCGTATTCT GAACGCCGCCGTCTTGAGCGATTGTGTAG
tolQ-mut-R	GGTGCAGAATCGCGGTGAACTCTTCCATAAAGTTGTCGTAATTCAGTTCCAGCTTGTTC ACGCGCTGATAATTAGCCATGGTCCATATG

Continued table S3

Primer	Sequence (5' to 3')
tolR-mut-F	ATCAATATTGTACCGCTGCTCGACGTACTGTTGGTGCTGCTGCTGATCTTTATGGCAACC GCGCCGATCCGTCTTGAGCGATTGTGTAG
tolR-mut-R	TACGCCCGCGCTGTGTAACAAGTTCAGCGCTTTAATTATTTCATCGTACGGCACCTCTTT CGCGCCGCCAATTAGCCATGGTCCATATG
tolB-Mtest-F	AGCAGGCATTACGAGTAGCA
tolB-Mtest-R	CAGATACGGCGACCAGGCAG
tolQ-Mtest-F	GTTCCTGAAGGCAAGCCTTC
tolQ-Mtest-R	TTTCGCTTACGGTAAACGCC
tolR-Mtest-F	CGAGGTCGTCGCGAACTTAA
tolR-Mtest-R	GATTGGCTGCGTCATTAAGC
tonB-compl-F	GCTAGCAGGAGGAATTCCATATGACCCTTGATTTACCTCGTC
tonB-compl-R	ATGCCTGCAGGTCGACTCTATTACTCAATCTGCGCCGTAC
exbB-compl-F	GCTAGCAGGAGGAATTCCATATGGGTAATAATTTGATGCAGACG
exbB-compl-R	ATGCCTGCAGGTCGACTCTATTACCCTACCCGTAATTTCTGC
exbD-compl-F	GGTTGTTTGGCACCGTATGG
exbD-compl-R	GCCAGTATTATCACAACGTCGT
tonB-Ctest-F	TGCGCTGCTTCTAAAGATTCAA
tonB-Ctest-R	ATGGATAATACTCCTCAGGGCG
exbB-Ctest-F	CGTAAAATAATTCGCCGTTGGC
exbB-Ctest-R	TTATTTCGCTTTCGCGGTCT
exbD-Ctest-F	GGTTGTTTGGCACCGTATGG
exbD-Ctest-R	GCCAGTATTATCACAACGTCGT
pBAD24-F	AGTGTCTATAATCACGGCAG
pBAD24-R	TTCACTTCTGAGTTCGGCAT
pagC-F	AGGCTTCCGGGTCTGTTG
pagC-R	TGTCGCCTTTACCGTGCC
sodC-F	TATCGGCACCGTCGTCAT

Continued table S3

Primer	Sequence (5' to 3')
sodC-R	GCAGCTACCGTTGGCATG
zntA-F	TTGAAGAGGCGGAAGAGCG
zntA-R	GCGGAATAAGCGTCACCAG
ackA-F	CACGGCGGCGAGAAGTAT
ackA-R	AGGTGAGCCGGGTTATGC
rpoD-F	ACATGGGTATTCAGGTAATGGAAGA
rpoD-R	CGGTGCTGGTGGTATTTTCA
rpsM-qpcr-F	GATCAGTGAGCTGTCTGAAG
rpsM-qpcr-R	ACCACGACGATGACGCAAAC
